# Intercomparison of Three Continuous Monitoring Systems on Operating Oil and Gas Sites

**DOI:** 10.1021/acsestair.4c00298

**Published:** 2025-03-18

**Authors:** William S. Daniels, Spencer G. Kidd, Shuting Lydia Yang, Shannon Stokes, Arvind P. Ravikumar, Dorit M. Hammerling

**Affiliations:** † Department of Applied Mathematics and Statistics, Colorado School of Mines, Golden, Colorado 80401, United States; ‡ Department of Petroleum and Geosystems Engineering, The University of Texas at Austin, Austin, Texas 78712, United States; ¶ Center for Energy and Environmental Resources, The University of Texas at Austin, Austin, Texas 78712, United States; § Energy Emissions Modeling and Data Lab, The University of Texas at Austin, Austin, Texas 78712, United States

**Keywords:** methane, oil and gas, intercomparison, continuous monitoring systems, emission mitigation, detection, localization, quantification

## Abstract

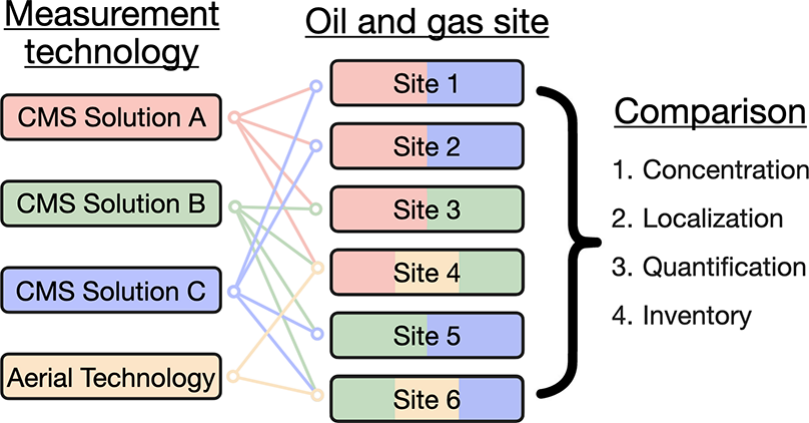

We compare continuous monitoring systems (CMS) from three different vendors on six operating oil and gas sites in the Appalachian Basin using several months of data. We highlight similarities and differences between the three CMS solutions when deployed in the field and compare their output to concurrent top-down aerial measurements and to site-level bottom-up inventories. Furthermore, we compare vendor-provided emission rate estimates to estimates from an open-source quantification algorithm applied to the raw CMS concentration data. This experimental setup allows us to separate the effect of the sensor platform (i.e., sensor type and arrangement) from the quantification algorithm. We find that 1) localization and quantification estimates rarely agree between the three CMS solutions on short time scales (i.e., 30 min), but temporally aggregated emission rate distributions are similar between solutions, 2) differences in emission rate distributions are generally driven by the quantification algorithm, rather than the sensor platform, 3) agreement between CMS and aerial rate estimates varies by CMS solution but is close to parity when CMS estimates are averaged across solutions, and 4) similar sites with similar bottom-up inventories do not necessarily have similar emission characteristics. These results have important implications for developing measurement-informed inventories and for incorporating CMS-inferred emission characteristics into emission mitigation efforts.

## Abstract 2

We compare three different continuous monitoring systems (CMS) on operating oil and gas sites over several months, with implications for CMS deployment in practice.

## Introduction

There has been a recent push toward the use of measurement-informed inventories for reporting methane emissions from the oil and gas sector, as traditional activity-based, bottom-up inventories have been shown to underestimate emissions.^1−4^ This move toward direct measurements has been reinforced by regulations in the United States,^5,6^ the European Union,^7^ and by global voluntary initiatives.^8^

Aerial survey-based measurement technologies (sometimes referred to as “snapshot” measurement technologies) are a common method for measuring methane concentrations and estimating emission rates. These technologies have been evaluated by many controlled release studies,^9−12^ showing reliable quantification accuracy with relatively well characterized error distributions.^13,14^ However, when using survey-based technologies, repeated measurements of any individual emission source are often separated by long periods of time (e.g., a three month gap between quarterly surveys). When surveys are conducted in this manner, the small number of measurements over time makes it challenging to accurately characterize the temporal variability of individual intermittent sources.^15,16^

Intermittency is now understood to be a feature of many types of emissions from the oil and gas sector.^17−21^ This has important implications for creating annualized inventories, as it means that a small number of measurements of an intermittent source may not capture the long-term average emission rate of that source. As such, survey-based technologies are often used to estimate large-scale (e.g., basin-scale) emission inventories, where the issue of intermittency is overcome by measuring a large number of sites.^22,23^ In this setting, it is reasonable to assume that emissions from a given site are distributed according to a common distribution shared by many other sites, and therefore, by measuring many sites, all possible emission states in their relative frequency will be observed.^24^ This assumption of a common emission distribution can be made at the basin-level or at smaller scales (e.g., by operator or site type).^25^

Continuous monitoring systems (CMS) measure site-level methane concentrations in near real time and therefore have the potential to create site-level inventories that account for highly intermittent emissions. These site-level inventories would require no assumptions about the emission characteristics of similar sites, as they would be based solely on measurements taken at the individual site level. Site-level inventories are required by the US Environmental Protection Agency (EPA),^6^ are important for differentiated gas markets at the sub-basin scale,^26,27^ and are required by voluntary initiatives like OGMP 2.0.^8^

Of course, the potential benefit of CMS-based, site-level inventories will only be realized if CMS solutions can be shown to accurately detect, localize, and quantify methane emissions. With this in mind, there is a growing body of literature aiming to both improve the capabilities of CMS and also evaluate their performance. Multiple teams are developing open-source methods for emission detection, localization, and quantification using CMS^28−31^ and for addressing CMS nondetect times when emitted methane is not captured by any sensor.^32^ These open-source tools can be used to benchmark private, proprietary solutions. On the evaluation side, CMS detection efficiencies and times to detection are being assessed using simulation studies,^33,34^ and multiple controlled release experiments have been conducted to evaluate the performance of different CMS solutions.^35−39^ Finally, there is a growing body of research evaluating CMS on operating oil and gas sites, as opposed to controlled release facilities, often by performing methane releases on top of the background emissions from normal operations.^40,41^

We add to this body of literature by comparing three point-in-space CMS solutions on six operating oil and gas sites in the Appalachian basin. No controlled releases were performed for this study, and as such, we have no ground truth to compare CMS results against. Instead, we compare CMS output to concurrent measurements from an aerial survey-based measurement technology and to site-level bottom-up inventories provided by the oil and gas operators. More broadly, we highlight the similarities and differences between the CMS solutions when deployed in the field.

Importantly, we directed the CMS solution vendors to deploy their sensors as they would in practice, rather than directing them to colocate all of their sensors. This was done intentionally to quantify the impact of different sensor deployments on emission source and rate estimates. Given this experimental setup, we add novelty to existing CMS evaluations by developing methodology to isolate the effect of the sensor platform (e.g., sensor type and arrangement) from the inversion method used to translate raw concentration measurements into source location and emission rate estimates. Results from this comparison highlight important considerations when using CMS to inform mitigation activities (e.g., site visits in response to alerts) and to create measurement-informed inventories at the site-level.

## Methods

### Description of Sites and Experimental Setup

Three different CMS solutions were deployed across six oil and gas sites in the Appalachian basin as a part of the Appalachian Methane Initiative (AMI). Two sites were compressor stations and the remaining four were production sites. The CMS were deployed in a pairwise comparison fashion, such that each site was instrumented with two different CMS solutions.

In addition to the CMS deployments, aerial measurements were conducted at hundreds of oil and gas sites as a part of the AMI project. The aerial measurement technology used in this study has an average 90% probability of detection emission rate of 1.27 kg/h.^42^ Two of the six sites instrumented with CMS were measured by the aerial technology. Table 1 lists the six sites with CMS, their type (production or compressor station), which CMS solutions were deployed, and if the site was overflown by the aerial technology. To address privacy concerns, the names of the CMS solutions, the aerial technology, and the oil and gas sites have been anonymized.

**Table 1:**
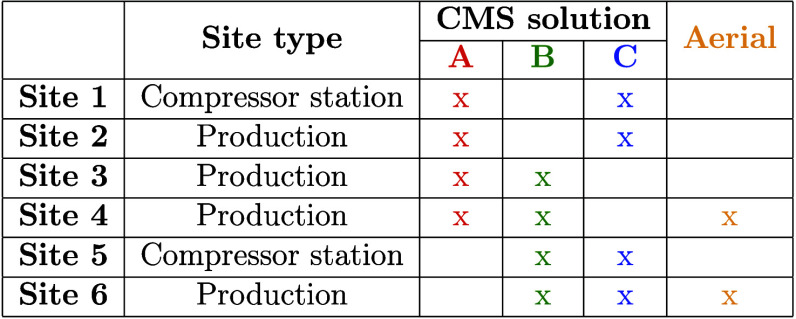
CMS Deployment Across the Six Oil and Gas Sites^a^

a The names of the CMS solutions and the oil and gas sites have been anonymized.

**Figure 1:**
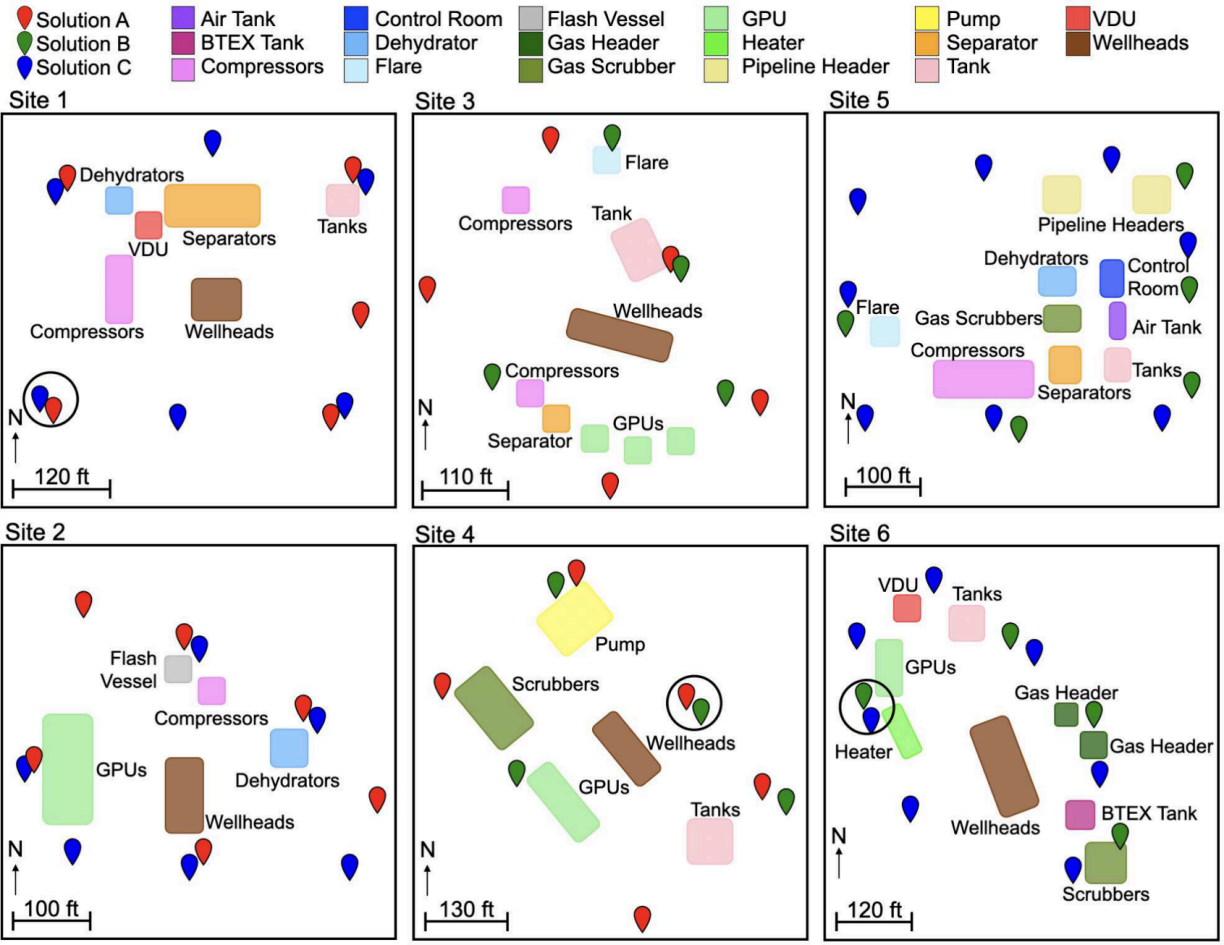
Schematics of the six oil and gas sites studied here. CMS sensor locations are marked with teardrop-shaped pins. Potential emission sources are marked with colored boxes. The closest two sensors for each combination of solutions across all six sites are circled in black.

The three CMS solutions studied here are point sensor networks, a class of CMS that leverages multiple fixed-in-space sensors (typically 4–8 per site) that measure methane concentrations in near real time. The sensors are typically arranged around the perimeter of the oil and gas site. A schematic of each site showing the location of the CMS sensors and potential emission sources is shown in Figure 1. This study evaluates the real-life deployment of CMS solutions, and as such, we directed the solution vendors to place their sensors according to their normal operating procedures. Details about each solution’s sensor placement procedure are proprietary and hence not known to the authors, but an open-source example is given in Jia et al.^43^ Critically, different sensor arrangements result in different estimates of emission source and rate. This was an intentional aspect of the study design, as we wanted to assess how the arrangement of CMS sensors impacts the emission estimates.

Table 2 lists a number of characteristics for each CMS solution. The CMS solutions did not have identical deployment periods. For each pairwise comparison between solutions (described below), we only use data from the subset of times in which both CMS solutions were deployed. Note that Solution B only reports a concentration measurement if it determines that a notable change has occurred from the last recorded measurement. The specifics of what constitutes a notable change are part of their propriety algorithms and are therefore not known to the authors. Also note that each CMS solution has their own proprietary algorithms for quantifying emission rate, which are not known to the authors.

**Table 2:**
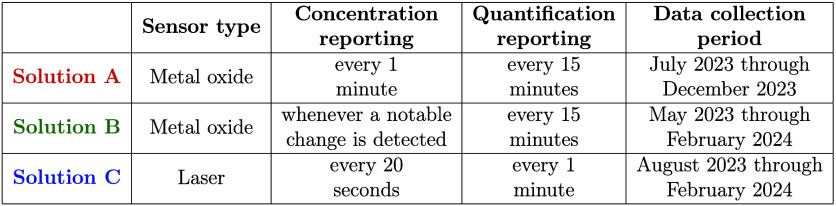
Characteristics of the Three CMS Solutions

### CMS Comparison Methodology

We perform six comparisons of the CMS solutions that each assess a different aspect of their capabilities. The methodology behind each comparison is discussed here, with results shown in the following section. Many of these comparisons use output from the open-source detection, localization, and quantification algorithm described below and in Daniels et al.,^31^ which we refer to as the “DLQ” algorithm.

### Concentration Measurements

CMS concentration measurements influence other inferred quantities (e.g., emission source and rate), and as such, we directly compare concentration data between CMS solutions. To account for the fact that none of the sensors are exactly colocated, we select the two closest sensors for each pair of CMS solutions for this comparison. The distances between these selected sensors are as follows: 2.83 m between solutions A and B, 10.03 m between solutions B and C, and 4.61 m between solutions A and C. The circled sensor pairs in Figure 1 are the sensors selected for this comparison. Because the sensor pairs are not exactly colocated, we limit our conclusions about concentration data to broad statements about their distribution, rather than specific differences in, e.g., the amplitude of a given concentration enhancement.

To provide as direct a comparison as possible, we transform the raw concentration data from each CMS solution such that the transformed data have one concentration observation per minute. For solution A, no transformation is required, as the raw concentration data are already provided at the minute-frequency. For solution B, we repeat each concentration measurement at the minute-frequency until a new observation is recorded. This is because solution B only records a new concentration observation when a notable change in the methane concentration time series is observed. Section S1 in the Supporting Information (SI) file discusses the potential implications of this upsampling procedure. For solution C, which reports concentration observations every 20 s, we average the three concentration observations that fall within a given minute, resulting in one concentration value per minute. Additionally, the DLQ algorithm takes concentration observations at the minute-frequency as input, so this transformation allows us to run the DLQ on the concentration data from each CMS solution.

To conduct the concentration comparison, we first plot a representative three hour period for each CMS solution pair to show detail in the concentration time series. For this plot, we selected time periods during which both of the CMS solutions being compared recorded elevated concentration enhancements (rather than background). Next, we plot the distribution of concentration measurements from the entire overlapping time period for each solution pair and compare the distribution means, widths, and empirical cumulative distribution functions.

Finally, to illustrate the characteristics of background-corrected concentration data, we repeat the comparison described above using background-corrected data. This comparison is shown in Section S2 of the SI file. The background correction procedure is as follows. First, we identify sharply elevated concentration values (“spikes”) using a gradient-based detection algorithm. These spikes are a result of emitted methane being blown toward the CMS sensors. We then cluster nearby spikes into groups, as there are often short gaps between spikes that are from small scale variability in wind direction rather than gaps in emissions. A separate background estimate is created for each group of spikes, which is taken to be the average of the concentration values immediately preceding and following the group. The background estimate for each group is then subtracted from all concentration values within the group. This procedure allows for hyper-local background estimates, as background concentrations are constantly fluctuating, and imposes no assumptions on the spatial homogeneity of the methane background. This background correction method is described in more detail in Daniels et al.^31^

### Localization Estimates

The ability to estimate emission source locations, referred to as “localization,” is an important aspect of CMS. For example, operators can use these estimates to determine if a site visit is necessary to fix a fugitive leak. However, not all of the CMS vendors involved in this study provided localization estimates. Therefore, we apply the DLQ algorithm to the minute-frequency concentration data from each CMS solution and compare the resulting localization estimates from this algorithm. The DLQ algorithm infers localization estimates by comparing the CMS concentration measurements to forward simulated concentrations from each possible source. The source whose simulated concentrations most closely match the actual concentration measurements (assessed using correlation) is selected as the localization estimate. Note that this procedure imposes the assumption that one source is emitting at a time. We use the Gaussian puff atmospheric dispersion model to forward simulate, which accounts for time-varying wind conditions and is described in detail in Jia et al.^44^

For each site, the DLQ algorithm is applied to the raw concentration measurements from each CMS solution. It is run on subsequent, nonoverlapping 30 min intervals, producing a localization estimate every 30 min for each solution. We slightly modify the DLQ algorithm as presented in Daniels et al.^31^ by omitting localization estimates for the 30 min intervals that have no concentration enhancements, as these intervals likely correspond to periods of no emissions. The length of the 30 min window was selected to balance the information content of each interval and the validity of the DLQ assumption that emission rate is constant within each interval. The 30 min interval was found to work well on a number of different sites.^16,31^ Additionally, the 30 min inversion interval used by the DLQ algorithm is comparable to what the CMS solution vendors use in practice, making it a natural choice for use in this study.

### Near Real Time Quantification Estimates

Estimating methane emission flux or rate, referred to as “quantification,” is important for near real time applications like prioritizing emission mitigation activities. We compare near real time quantification estimates provided by the CMS vendors (using their proprietary algorithms) and from the DLQ algorithm.

The DLQ algorithm infers an emission rate estimate by minimizing the mean squared error between simulated concentrations from the most likely source and the actual CMS concentration measurements by scaling the amplitude of the simulated concentrations. The emission rate that minimizes error is taken to be the emission rate estimate. We run this algorithm on the minute-frequency concentration data from each solution separately in subsequent, nonoverlapping 30 min intervals. This provides a rate estimate every 30 min for each CMS solution. Note that this procedure imposes the assumption that one source is emitting at a constant rate within each 30 min interval. We create confidence intervals for the emission rate estimates by bootstrapping the available data within each 30 min interval, which creates a distribution of possible emission rates.

The CMS vendors provide quantification estimates at either a 15 min frequency (solutions A and B) or a 1 min frequency (solution C). Therefore, to compare individual rate estimates between CMS solutions, we must average the vendor-provided rate estimates onto the same temporal frequency. Failing to do so would result in a comparison of rate estimates that were based on different time periods, and hence emission intermittency would introduce additional variability between the CMS solutions. To better align with the rate estimates from the DLQ algorithm, we average the vendor-provided rate estimates up to a 30 min frequency.

To perform the near real time comparison of these rate estimates, we first align them in time (so that they are based on the same 30 min) and then plot them against each other in a parity plot. We compare vendor-provided estimates against vendor-provided estimates and DLQ estimates against DLQ estimates.

This methodology allows us to isolate the effect of the quantification algorithm from the sensor platform itself (i.e., the sensor type and arrangement). The comparison between vendor-provided estimates is subject to differences in the sensor type (i.e., metal oxide vs laser), the number and arrangements of sensors, and the quantification algorithm used to produce rate estimates. We effectively control for the impact of the quantification algorithm by running the same open-source DLQ algorithm on the raw data from each solution. Thus, the resulting differences in the DLQ rate estimates are due solely to the sensor platform.

### Quantification Estimates in Distribution

Quantification estimates are also useful when studied in distribution, as long-term aggregates of quantification estimates can be used for annualized emissions reporting at the site-level. To compare quantification estimates in distribution, we bin the vendor-provided quantification estimates (averaged up to a 30 min frequency) and the estimates from the DLQ algorithm. Similar to the near real time quantification comparison, we compare vendor-provided estimates against vendor-provided estimates and DLQ estimates against DLQ estimates. Note that this procedure again allows us to separate the influence of the sensor platform and the quantification algorithm. Comparisons are made using distributional averages and empirical cumulative distribution functions. Confidence intervals for the distributional averages are calculated by randomly sampling 50% of the distribution 1,000 times, taking the mean of each sample, and then selecting the 0.025 and 0.975 quantiles as the 95% interval.

### Comparison to Aerial Data

Having no ground truth controlled release data to compare CMS estimates against, we instead compare to rate estimates from an aerial measurement technology with well documented quantification performance. To perform this comparison, we first identify the 30 min quantification interval that overlaps with each overpass of the aerial technology. If multiple overpasses occur during a given 30 min interval, we average the aerial estimates so that there is one value to compare the CMS output against. We then compare the CMS quantification estimates during that interval (both from the vendor and from the DLQ algorithm) to the estimated emission rate from the aerial technology. The aerial technology measured Site 4 five times and Site 6 two times, providing seven measurements to compare against, and did not measure any of the other four sites. 95% confidence intervals on the individual rate estimates from the aerial technology are computed using the error distributions from Conrad et al.^14^

### CMS-Based Inventory

We create and compare four versions of a CMS-based measurement-informed inventory for each site. The first two inventories are created using the vendor-provided quantification estimates averaged up to a 30 min frequency. The second two are created using the rate estimates from the DLQ algorithm applied to the minute-frequency concentration observations from the two CMS solutions on the site. For each site, we compare the four CMS-based inventories to a site-level bottom-up inventory prepared according to emissions reporting guidelines set by the Pennsylvania Department of Environmental Protection^45^ and the US EPA.^46^

We create the CMS-based inventories using the same methodology, regardless of the source of the emission rate estimates. We first sum all of the rate estimates across the entire time period in which there is overlapping data from the two CMS solutions being compared. We then normalize the inventory to the month-scale by dividing the sum by the number of days within the overlapping time period and multiplying by 30 (i.e., assuming a 30-day month).

The DLQ algorithm provides a distribution of possible emission rates for each 30 min quantification interval as a measure of uncertainty. This allows us to quantify uncertainty in the inventories based on these estimates by using a Monte Carlo framework. Specifically, for each iteration of the Monte Carlo framework, we take one sample from the distribution of possible emission rates for each 30 min interval, sum the resulting samples, and scale the sum to a 30-day inventory. This provides one possible inventory value for the site. By repeating this many times, we build a distribution of possible inventory values. When presenting these results, we show the mean and 95% interval of these distributions.

## Results

### Concentration Measurements

**Figure 2:**
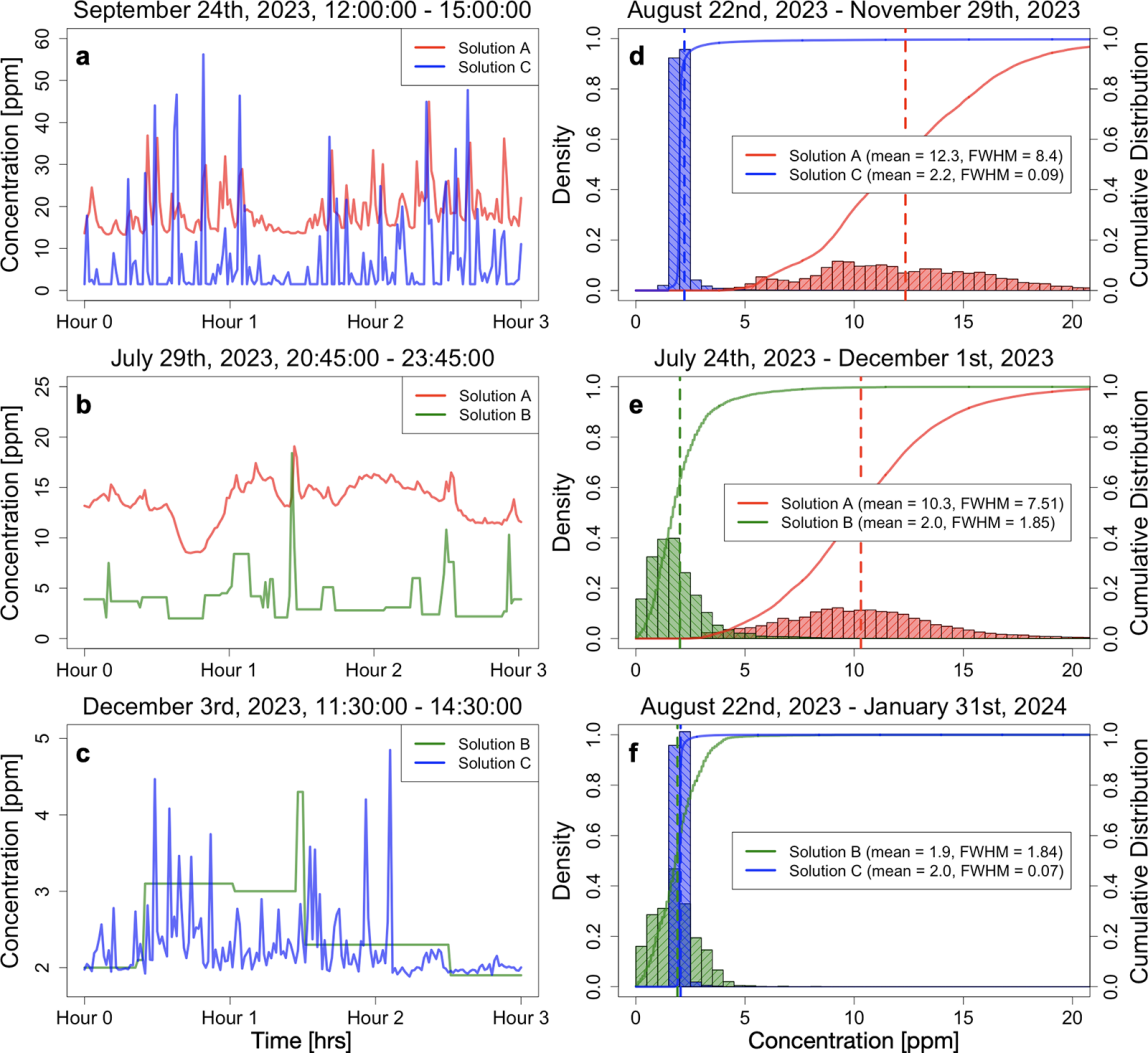
Concentration data from the nearly colocated sensor pairs, with each row showing data from a different sensor pair. See Figure 1 for the location of each sensor pair shown here. (a)-(c) zoom in on a representative three hour period to show detail. Note that (a)-(c) have different vertical scales, as they show data from different sites and time periods and hence are not meant to be directly compared. (d)-(f) show the distribution of concentration measurements from the entire time period during which both solutions were deployed. Solid lines show the empirical cumulative distribution functions, and vertical dashed lines show the distribution average. Full width half-maximum (fwhm) values are listed for each solution.

Figure 2 shows concentration data from the nearly colocated sensor pairs circled in Figure 1. Subfigures (a)-(c) show a representative 3-h period to highlight detail, and subfigures (d)-(f) show the distribution of concentration measurements from each sensor during their entire overlapping time period. Section S5 in the SI contains Quantile-Quantile (QQ) plots showing the distribution of concentration observations plotted in Figure 2.

The average concentration measurements from solution A (12.3 and 10.3 ppm) are much larger than the average concentration measurements from solution B (2.0 and 1.9 ppm) and solution C (2.2 and 2.0 ppm). We expect the average concentration measurement from these sensors to be close to the local background concentration for this region, which is between 1.9 and 2.1 ppm.^47^ Therefore, the concentration measurements from solution A, which uses metal oxide sensors, are likely biased high.

Solution B only reports a new concentration value if a large enough change in the concentration time series is detected. As a result, these data have many periods of constant concentration measurements. Despite also using metal oxide sensors, solution B does not have a large positive bias in their concentration data, with distributional means close to the expected background value.

The distributions of concentration measurements from the two solutions that use metal oxide sensors (solutions A and B) are wider than the distribution of concentration measurements from solution C, which uses laser-based sensors. Specifically, full width half-maximum (fwhm) values are 8.4 and 7.5 ppm for solution A and 1.85 and 1.84 ppm for solution B, while they are only 0.09 and 0.07 ppm for solution C.

Despite the different characteristics mentioned above, all CMS solution pairs largely observe concentration enhancements at the same time, as seen in subfigures (a)-(c). Certain sensor characteristics (i.e., a positive bias and wider distribution) can be corrected for when, e.g., estimating emission rate, but false negative or false positive enhancements would result in under- or overestimated emissions, respectively.

Finally, we note that the CMS solution vendors likely account for the various characteristics of their raw concentration data when, e.g., producing localization and quantification estimates. When using the raw data directly, however, it is important to be aware of these characteristics, as they could influence inferred quantities if not properly addressed. For example, it is necessary to remove the (likely overestimated) background from solution A’s concentration data to accurately estimate emission rates using these data. Figure S2 in the SI file shows the same concentration data as in Figure 2, but background-corrected using the DLQ algorithm.

### Localization

**Figure 3:**
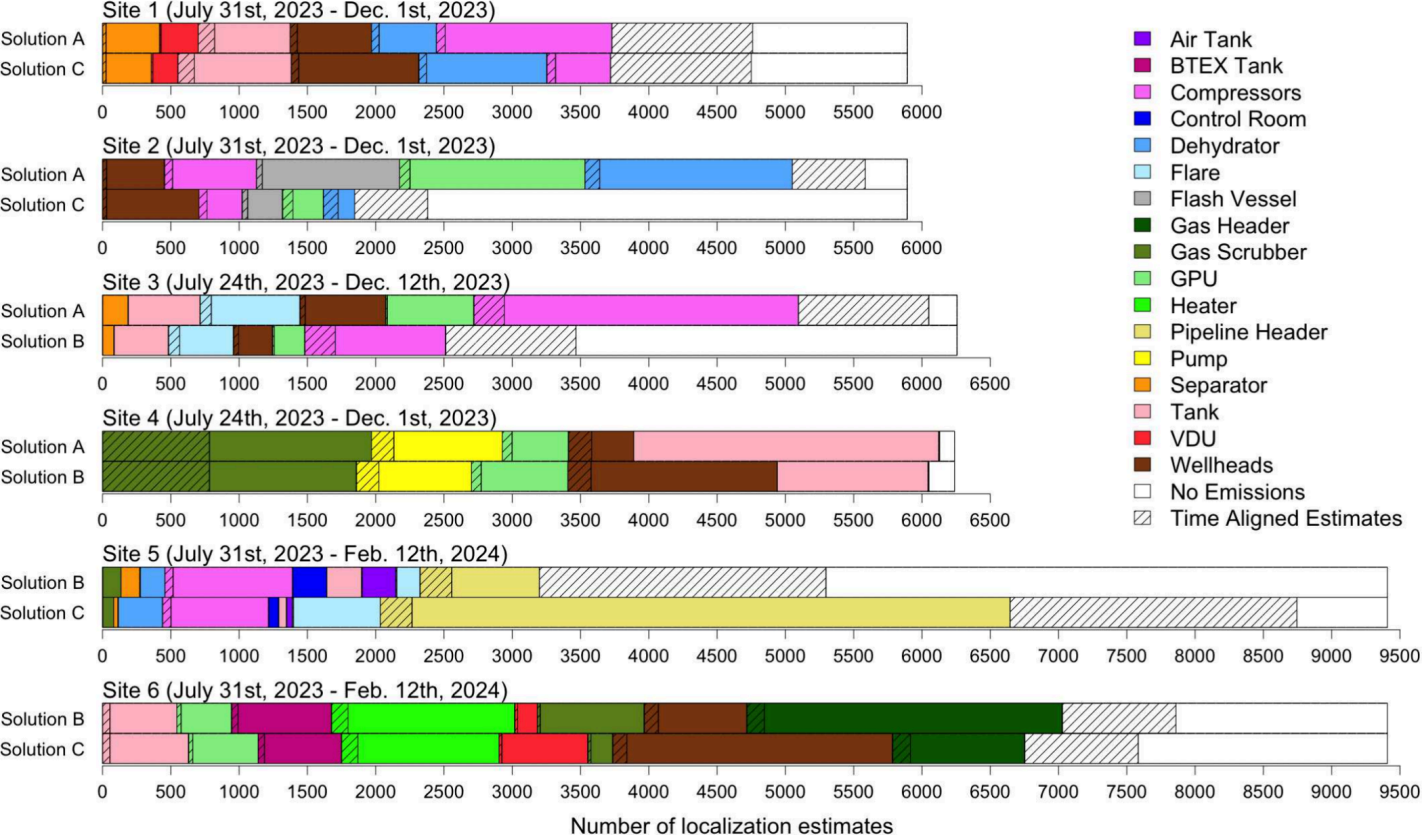
Localization estimates from the open-source DLQ algorithm across the six sites included in this study. For each site, the two bars show the localization estimates from the two solutions installed on that site. Colors correspond to the source estimates, and cross-hatched regions indicate localization estimates that were made at the same time between the two solutions installed on the site.

Localization estimates from the open-source DLQ algorithm are shown in Figure 3. The amount of time when both CMS solutions were deployed varied by site, and as such, the total number of localization estimates varies by site. For each site, we group the localization estimates by source and order them by the magnitude of the difference between the two solutions. A cross-hatched pattern shows the subset of estimates for a given source that were made at the same time between the two solutions. The information contained in Figure 3 is given in Section S3 in the SI in table format for more detailed analysis.

We discuss two forms of alignment between the CMS solution pairs: alignment in time and in distribution. Alignment in time is defined as the percent of localization estimates that are the same between CMS solutions at the same time. In other words, it is the percent of 30 min localization intervals for a given site that have the same localization estimate between CMS solutions. Visually, this is the percent of the bars in Figure 3 that have the cross-hatch pattern. Alignment in time can be interpreted as near real time agreement between the two solutions, as this quantifies the amount of individual 30 min intervals in which the localization estimates agreed; a matter that is pertinent for near real time applications like alerting. Alignment in distribution is defined as the percent of localization estimates that are the same between CMS solutions when aggregated over the entire study period, regardless of if the estimates occurred at the same time. Visually, this is the percent of the bars in Figure 3 that have overlapping colors, including both the cross-hatched periods and the not cross-hatched periods. Alignment in distribution can be interpreted as long-term agreement between the solutions when aggregated at the month-scale. This is more pertinent for annualized applications, like inventory reporting. Alignment numbers are provided in Table 3, and an example calculation for both metrics is given in Section S3 in the SI.

**Table 3:**
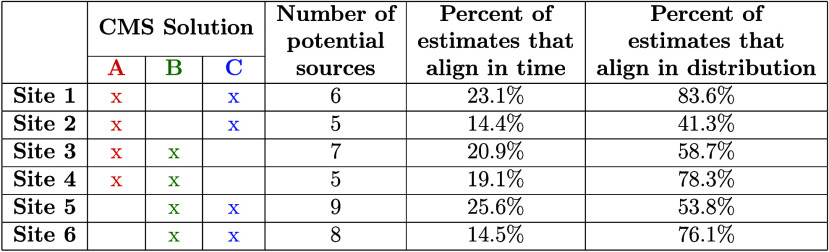
Percent of Localization Estimates That Align in Time and in Distribution for Each Site^a^

a Alignment in time refers to estimates that are the same between CMS solutions at the same time. Alignment in distribution refers to estimates that are the same between solutions after being aggregated (i.e., when ignoring the time of the estimate and only looking at the number of estimates per source).

There is a high degree of variability in near real time localization estimates, but broad agreement when aggregated at the month-scale (or longer). Specifically, alignment in time ranges from 14.4% to 25.6% across sites (average = 19.6%), while alignment in distribution ranges from 41.3% to 83.6% across sites (average = 65.3%). This has important practical implications for alerting, namely that individual localization estimates may not be reliable and longer time aggregates should be utilized. For example, a rolling mode could be used to inform leak mitigation activities, where the most common source over a, e.g., 4 h window could be used to identify leak sources rather than individual 30 min estimates.

Note that variability in inferred quantities (e.g., localization estimates) over short time scales does not necessarily diminish the usefulness of CMS. However, this variability must be carefully accounted for when using CMS to inform emissions reporting or leak mitigation activities. We further note that variability on these short time scales could be the result of errors in the forward dispersion model that average out when taken in aggregate over longer periods.

Finally, we do not see a relationship between site complexity (assessed by the number of potential emission sources) and alignment in time between CMS solutions. Some complex sites have low alignment (Sites 3 and 5), as one might expect, but others have high alignment (Site 6). Similarly, some simple sites have high alignment (Sites 1 and 4), as one might expect, but others have low alignment (Site 2). Ultimately, however, a larger sample of sites is needed to draw any definitive conclusions about the effect of site complexity on localization performance.

### Near Real Time Quantification

**Figure 4:**
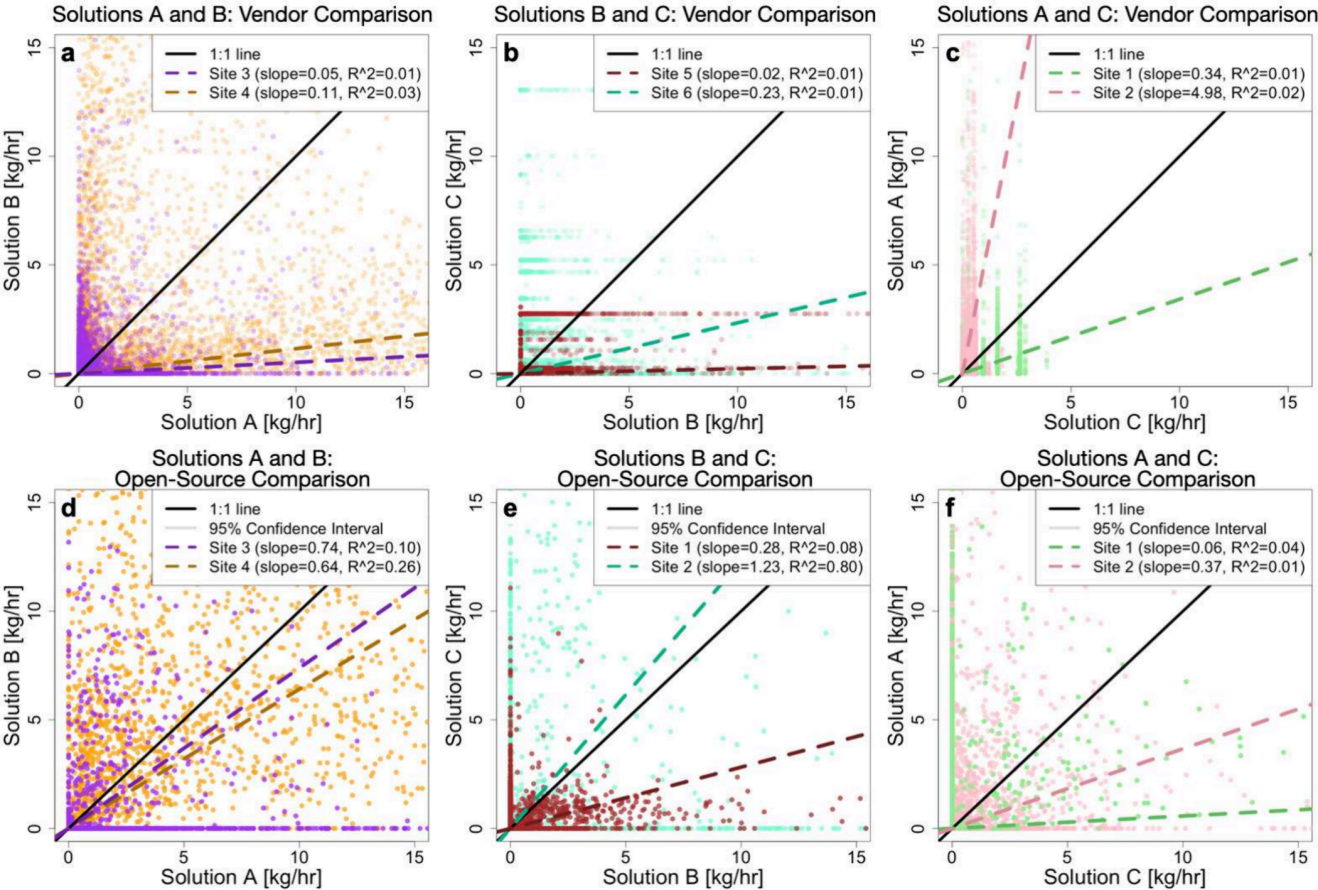
Parity plots comparing emission rate estimates made at the same time by the different CMS solutions. (a)-(c) compare rate estimates provided by the CMS vendors, and (d)-(f) compare rate estimates from the open-source DLQ algorithm applied to the raw concentration data from each CMS solution. Each point shows two rate estimates produced during one 30 min quantification interval. Each subfigure uses data from the two oil and gas sites that have the two solutions installed (see Table 1). Axes are restricted to [0, 15] kg/h to show detail.

Figure 4 compares time-aligned emission rate estimates, with subfigures (a)-(c) showing vendor-provided rates and subfigures (d)-(f) showing rate estimates from the DLQ algorithm. Solution C produces emission rate estimates at discrete values, resulting in horizontal lines in subfigure (b) and vertical lines in subfigure (c). Figure 4 does not include confidence intervals, as there are too many data points to match a given confidence interval to its corresponding rate estimate. For completeness, however, a version of Figure 4 that includes confidence intervals is included in Section S4 of the SI.

There is very poor alignment between emission rate estimates at the 30 min time scale. Slopes of the best fit lines range from 0.02 to 4.98 in the vendor comparisons and from 0.06 to 1.23 in the open-source comparisons. R^2^ values range from 0.01 to 0.03 for the vendor-provided estimates and from 0.01 to 0.80 for the open-source estimates. Differences in the vendor comparisons, subfigures (a)-(c), could be caused by the sensor platform (i.e., sensor type and arrangement) or the proprietary quantification algorithm used to translate the raw concentration data into emission rate estimates. The open-source comparisons, subfigures (d)-(f), control for the quantification algorithm, meaning that differences in these comparisons are due to the sensor platform. Therefore, a large improvement in the alignment of the open-source comparison compared to the vendor comparison indicates that much of the differences in the vendor comparison were due to the quantification algorithm. Conversely, a small improvement (or no improvement) in the alignment of the open-source comparison compared to the vendor comparison indicates that much of the differences in the vendor comparison were due to the sensor platform.

When moving from the vendor to the open-source comparisons, there is a larger improvement in the alignment of the solution A-B and solution B-C slopes than the solution C-A slopes. This implies that the quantification algorithm has a larger impact on the differences between solutions A and B and solutions B and C than it does on the differences between solutions C and A.

Overall, poor alignment in the near real time quantification estimates, even after controlling for the quantification algorithm, indicates that near real time quantification estimates from CMS currently have large uncertainties, as differences in sensor platform can cause dramatic differences in emission rate estimates. This has important practical implications for alerting, namely that individual quantification estimates may not be reliable and longer time aggregates (e.g., multihour averages) should be utilized for prioritizing mitigation activities. Furthermore, alerts based on longer term aggregates may be better suited for detecting fugitive emissions due to, e.g., leaks or stuck dump valves.

### Quantification in Distribution

**Figure 5:**
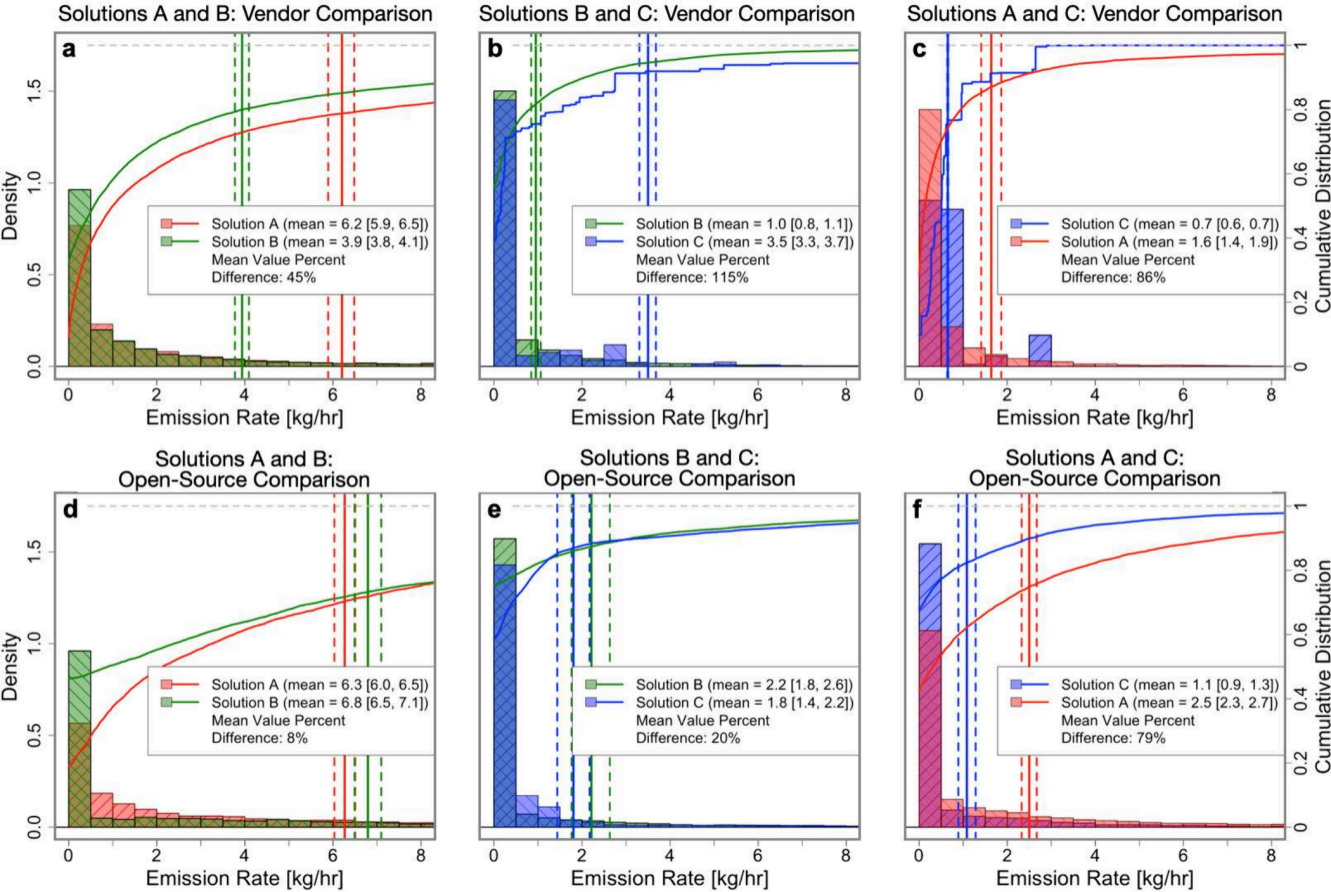
Distribution of emission rate estimates for each CMS solution pair. (a)-(c) show rate estimates provided by the CMS vendors, and (d)-(f) show rate estimates from the open-source DLQ algorithm applied to the raw concentration data from each CMS solution. Each subfigure uses data from the two oil and gas sites that have the two solutions installed (see Table 1). Solid lines show empirical cumulative distribution functions, solid vertical lines show distribution averages, and dashed vertical lines show 95% confidence intervals for the averages. Horizontal axes are restricted to [0, 8] kg/h to show detail. Density is a scaled version of the counts in each bin such that each histogram has a unitary area.

Figure 5 compares emission rate estimates in distribution, with subfigures (a)-(c) showing vendor-provided rates and subfigures (d)-(f) showing rate estimates from the DLQ algorithm. Note that each comparison (i.e., each subfigure) uses data from both of the sites that have the given solutions installed. To supplement Figure 5, QQ plots of these emission rate distributions are provided in Section S5 of the SI.

The distribution of CMS emission rate estimates are similar in shape, despite poor alignment between individual estimates at the 30 min scale. Broadly speaking, this means that different CMS solutions might disagree at any one point in time, but agree (to varying degrees) when their rate estimates are aggregated over time. Specifically, the differences between distribution averages range from 0.9 to 2.5 kg/h for the vendor comparisons, subfigures (a)-(c), and from 0.4 to 1.4 kg/h for the open-source comparisons, subfigures (d)-(f). To probe how the alignment between CMS solutions changes as the length of the aggregation period increases, Section S6 in the SI contains a similar analysis using different aggregation periods ranging from the 30 min intervals used in Figure 4 to the entire time series used in Figure 5.

As with the near real time analysis, differences in the temporally aggregated rate estimates from the solution vendors could be the result of the sensor platform (i.e., sensor type and arrangement) or the quantification algorithm, while differences in the open-source rate estimates are only from the sensor platform. This allows us to disentangle the effect of the sensor platform and the quantification algorithm by comparing the magnitude of the differences between the vendor comparisons and the open-source comparisons.

For the solution A-B and solution B-C comparisons, the percent difference in distribution average decreases notably from the vendor comparison to the open-source comparison. This implies that much of the difference in aggregated quantification estimates between solutions A and B and solutions B and C comes from the quantification algorithm, as the average quantification estimate aligns closely after controlling for the quantification algorithm. For the solution C-A comparison, however, the percent difference in distributional average decreases only slightly from the vendor comparison to the open-source comparison. This implies that much of the difference in aggregated quantification estimates between solutions C and A comes from the sensor platform, not the quantification algorithm.

These results suggest that quantification estimates taken in aggregate can be partially reconciled between CMS solutions by controlling for the algorithm used to estimate emission rates. This is promising, as long-term averages of CMS-based quantification estimates can be used to create measurement-informed methane inventories that capture site-level intermittency if they are shown to be reliable through repeated evaluation. However, given that only six sites were included in this study, an analysis of more sites is necessary to both verify and generalize these findings to different sites.

### Comparison to Aerial Data

**Figure 6:**
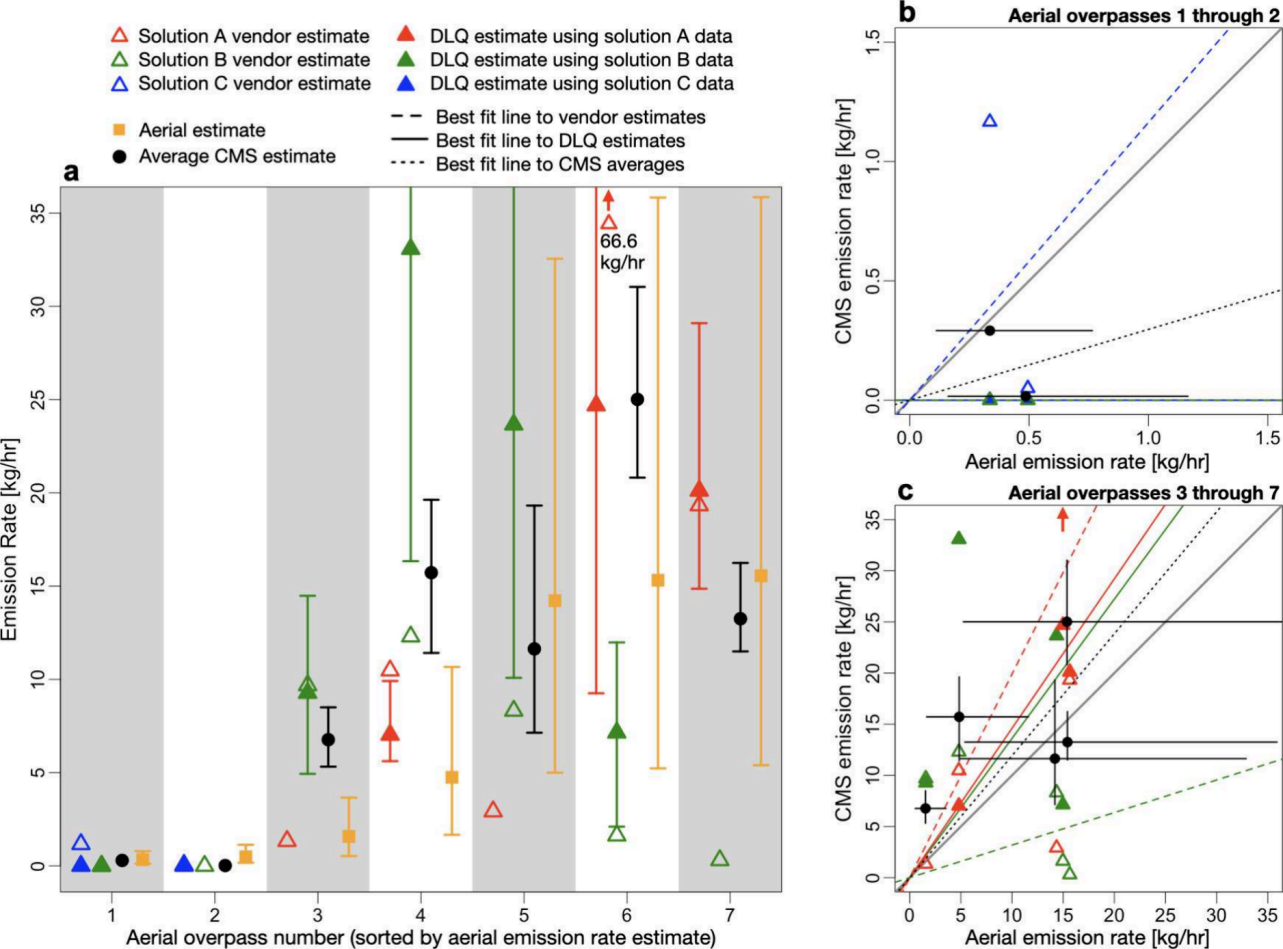
Comparison of the CMS emission rate estimates to rate estimates from the aerial technology. Uncertainties are 95% confidence intervals. (a) shows the rate estimate from each aerial overpass and the CMS rate estimates from the coinciding 30 min quantification interval. (b) and (c) show parity plots of the CMS rate estimates and the aerial estimates for the two overpasses on Site 6 and the five overpasses on Site 4, respectively. Dashed lines show best fit lines to the vendor-provided rate estimates, solid lines show best fit lines to the DLQ rate estimates, and the dotted lines show the best fit lines to the average of all CMS rate estimates.

Figure 6 compares CMS rate estimates to estimates from the aerial technology. The aerial technology conducted 7 overpasses of sites instrumented with CMS, providing 7 instances for comparison. Overpasses 1 and 2 occurred on Site 6, and overpasses 3 through 7 occurred on Site 4. Note that there is a very limited sample of aerial measurements for comparison, and as such, further work is needed to assess the generalizability of the results in this section.

The DLQ algorithm uses Monte Carlo sampling to create a distribution of possible emission rates for each 30 min interval. We construct 95% confidence intervals for the DLQ estimates as the inner 95% interval of these distributions. Vendor-provided CMS rate estimates did not come with an associated uncertainty estimate, and hence no confidence intervals are shown for these estimates. We construct 95% confidence intervals for the aerial estimates using the error distributions from Conrad et al.^4^

Overpasses 1 and 2 on Site 6 both detected small emissions (<1 kg/h). Corresponding rate estimates from the DLQ algorithm and solution C were all zero (indicating no emissions), while estimates from solution B were nonzero but still small. While the number of overpasses in the small emission rate regime is limited, these two instances provide some evidence that CMS are able to identify periods of small or no emissions.

Overpasses 3 through 7 on Site 4 primarily detected larger emissions (>1 kg/h). As discussed in the near real time quantification section, CMS-based rate estimates at the 30 min scale vary widely between CMS solutions and quantification algorithms (see Figure 4). This is also seen in Figure 6, with CMS rate estimates ranging from −198% to 526% of the corresponding aerial rate estimates on this site. Averaging the CMS rate estimates across solutions and quantification algorithms results in closer agreement with the aerial estimates, with CMS averages ranging from −25% to 292% of the aerial estimates on this site.

Figures 6(b) and 6(c) show parity plots comparing the CMS rate estimates to the aerial estimates. A very small number of measurements on Site 6 (overpasses 1 and 2) make it challenging to draw any conclusions. Slightly more measurements at higher emission rates makes a comparison more feasible on Site 4 (overpasses 3 through 7). Rate estimates provided by solution A tend to overestimate the aerial estimates (slope = 1.99), while rate estimates provided by solution B tend to underestimate the aerial estimates (slope = 0.32). Rate estimates from the DLQ algorithm are more closely aligned with the aerial estimates (i.e., best fit lines closer to parity) but still exhibit positive bias, with a slope = 1.46 for solution A and a slope = 1.36 for solution B. Averages across all CMS rate estimates, both from the vendors and the open-source algorithm, are closest to parity (slope = 1.19). Averaging multiple estimates dampens variability, so this result is not unexpected. However, this finding is still promising, as it shows that the average of multiple CMS-based rate estimates is converging toward the estimates from the aerial technology. This suggests that the CMS estimates are coming from similar emission distributions, which is in line with results from Figure 5 (especially for the solution A-B comparison).

### CMS-Based Inventory

**Figure 7:**
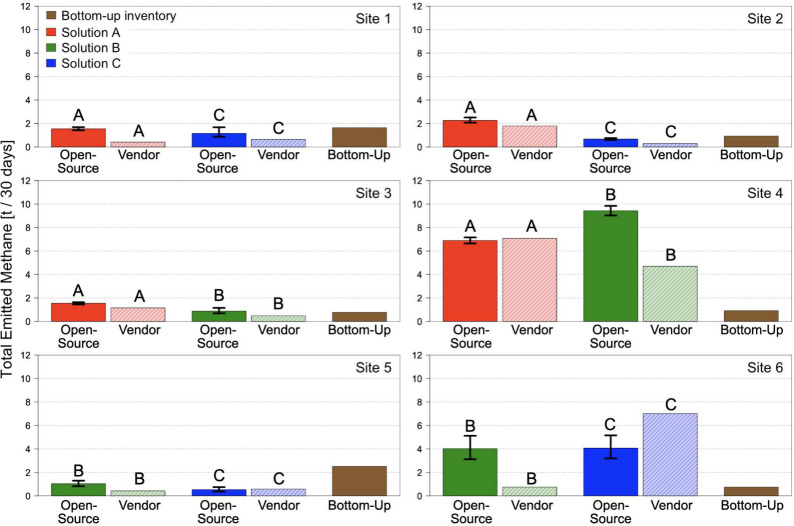
Site-level, measurement-informed methane inventories created using CMS data. Solid bars labeled “Open-Source” show inventories created using rate estimates from the DLQ algorithm applied to the raw concentration data from the CMS solutions. Lighter, cross-hatched bars labeled “Vendor” show inventories created using the vendor-provided rate estimates. The letters “A”, “B”, and “C” above “Open-Source” and “Vendor” bars denote the solution the respective inventory comes from. Brown solid bars labeled “Bottom-Up” show the site-level bottom-up inventories provided by the oil and gas operators. Black lines show 95% confidence intervals. Each inventory is normalized to 30 days.

Figure 7 shows the CMS-based measurement-informed inventories for each site. A separate inventory value is computed for each CMS solution and quantification algorithm, resulting in four inventory values per site. Emission rate estimates from the DLQ algorithm have associated uncertainty, allowing for 95% confidence intervals on the inventories produced using these estimates. The vendor-provided estimates did not have an associated uncertainty, and hence no uncertainty is provided for the inventories created using these estimates. CMS-based inventories are compared against site-level bottom-up inventories provided by the oil and gas operators.

CMS-based inventories broadly agree between solutions and quantification algorithms, with all sites except for Site 2 having an average percent difference of less than 50% between the four CMS-based inventories and their average. In particular, Sites 1, 2, 3, and 5 have inventory values around or below 2 t/30 days and Sites 4 and 6 have inventory values primarily above 2 t/30 days. This finding is in line with results from the previous sections showing that CMS rate estimates tend to disagree dramatically at the 30 min scale (i.e., Figure 4) but more closely agree in distribution over longer time periods (i.e., Figure 5).

Comparison to bottom-up inventories reveals that similar oil and gas sites do not necessarily have similar emission characteristics. Sites 2, 3, 4, and 6 are all production sites with five to eight equipment groups and bottom-up inventories ranging from 0.75 to 0.93 t/30 days. Despite these similarities, the average across CMS-based inventories is 7.69× and 5.28× larger than the bottom-up inventories for Sites 4 and 6 but only 1.35× and 1.32× larger for Sites 2 and 3. This suggests that bottom-up inventories are not always able to capture emission variability between sites, a finding in line with existing literature (e.g., Wang et al.^21^). CMS-based inventories are all slightly smaller than the bottom-up inventories for the two compressor stations (Sites 1 and 5), but the limited sample of compressor stations makes it challenging to generalize this conclusion.

## Discussion

### Primary Findings and Comparison to Literature

This study has revealed a number of important considerations for deploying CMS in practice on oil and gas sites. First, care should be taken when interpreting raw CMS concentration data, as these data have different characteristics depending on the sensor type and CMS solution. We find that concentration data from metal oxide sensors have higher variability than data from laser-based sensors and can have large positive bias compared to the expected methane background of 1.9 to 2.1 ppm for the study region. Different background offsets in concentration data between CMS sensors was also found in Yang and Ravikumar^41^ and Day et al.,^40^ with Yang and Ravikumar^41^ noting that the correlation between concentration data from colocated CMS sensors was slightly higher when they were background-corrected.

Second, there is a high degree of variability in both localization and rate estimates at the 30 min scale using the CMS solutions studied here. These near real time estimates are often used by oil and gas operators to determine when a site visit is necessary to mitigate methane emissions. We find that using longer term aggregates (e.g., multihour averages or maximums) will likely reduce false positive alerts and provide more meaningful information to optimize the deployment of emission mitigation personnel. This finding aligns with the latest controlled release evaluation presented in Cheptonui et al.,^39^ which found that many solutions had high variability in their individual rate estimates.

Third, localization and quantification estimates from the CMS solutions have similar distributions when aggregated over multiple months. This means that applications on longer time scales (e.g., annual inventories) are less sensitive to the type of CMS sensor deployed. It is important to note that we performed no controlled releases in this study, and therefore we are unable to assess if these distributions are converging to the truth. Continued evaluation of CMS solutions in a controlled release setting is necessary to evaluate their performance relative to a known ground truth. This finding again aligns with the most recent controlled release evaluation presented in Cheptonui et al.,^39^ which found that, despite high variability in individual emission rate estimates, the better performing CMS solutions had minimal bias in their emission rate estimates. This means that long-term emission rate averages from these solutions will approach the true emission rate. It is important to conduct studies like Day et al.,^40^ Yang and Ravikumar,^41^ and this work alongside controlled release evaluations to monitor the performance of CMS when deployed in practice.

Finally, comparing CMS-based measurement-informed inventories to bottom-up inventories reveals that similar oil and gas sites do not necessarily have the same emission characteristics. CMS-based emission estimates were closely aligned with bottom-up inventories on two of the four production sites studied here, but were much larger than bottom-up inventories on the remaining two production sites. This variability in emission volume between similar sites underscores the importance of repeated site-level measurements when creating measurement-informed inventories at the site-level, as aggregating survey-based measurements across similar sites may fail to capture large differences in emissions.

### Policy Implications

Findings in this study have important implications for regulatory compliance as specified in the recently finalized EPA methane rule.^48^ The rule specifies two sets of criteria for the use of CMS as part of leak detection and repair programs: a detection criteria and an action criteria. The detection criteria requires CMS to be able to detect emissions below 0.4 kg/h and report a site-level emission rate at least once every 12 h. The action criteria, which varies by the type of oil and gas facility, requires operator follow-up action based on emissions exceeding set thresholds over a 7- or 90-day rolling period above site-specific emissions baseline.

Our analysis suggests that the action criteria requiring aggregation of CMS-based quantification estimates across several days or months is likely to provide reasonably accurate results for appropriate follow-up action. However, because these action thresholds are relative to site-specific baseline emissions, whether CMS technologies can consistently identify long-term excursions from baseline will also depend on the magnitude of baseline emissions. For example, an average excursion of 1.2 kg/h over a 90-day period (well-site action threshold) against a baseline of 0.3 kg/h is qualitatively different from an average excursion of 1.6 kg/h (compressor station action threshold) against a baseline of 10 kg/h. Recent research suggests that CMS may be more effective in the former scenario than in the latter.^41^

Poor agreement between CMS solutions on short time scales (i.e., 30 min) indicates that individual emission rate estimates near the 0.4 kg/h detection threshold will likely have high variability. However, this study is poorly suited to assess the ability of CMS to meet the 0.4 kg/h detection threshold, as we focused on the ability of CMS to localize and quantify emissions over their ability to detect emissions. Poor agreement on individual emission rates between CMS solutions does not necessarily mean that CMS cannot detect small emissions, just that the estimated emission rates will have high variability. Controlled release evaluations are necessary to assess the minimum detection limits of CMS, with recent studies indicating that some CMS solutions are approaching the 0.4 kg/h threshold.^39^

Irrespective of the short-term agreement between CMS solutions, temporal aggregation over several hours to weeks will likely provide actionable information to operators to help identify and mitigate abnormal emission events, especially when compared to instantaneous concentration readings or short-term (e.g., 30 min) quantification estimates.

### Key Assumptions and Limitations

We conclude with a number of important considerations. First, this work includes only six oil and gas sites. We do not claim that these results would be perfectly replicated on different sites and under different conditions. However, we believe that this analysis, although limited to six sites, provides an important step toward understanding real world CMS performance. Future work will include an analysis of CMS on a larger sample of sites. Second, the exact degree of near real time quantification alignment is likely a function of the length of the quantification interval used in the inversion. Performing the inversion on longer intervals would likely result in more near real time agreement, and vice versa. However, the amount of data used to infer emission source and rate was not the primary focus of the manuscript, and we believe that using a comparable interval to what CMS solutions are using in practice most genuinely reflects the real world performance of CMS. Third, differences in the near real time and aggregated emission rate estimates between the CMS solutions are partially due to differences in the localization estimates. Section S7 in the SI explores this source of variability by examining near real time and distributional agreement between CMS solutions for 30 min intervals where the localization estimates from each solution were the same.

Finally, it is important to restate the primary assumptions of the DLQ algorithm. Within a given 30 min interval, the algorithm assumes a single source is emitting at a constant rate. This could impact the accuracy of the quantification estimates on the more complex sites where more than one source could be emitting at a time. It could further impact the comparison to aerial measurements and bottom-up inventories, which are better able to accommodate multisource emissions. Additionally, the DLQ algorithm, and possibly the proprietary algorithms used by the CMS solutions, employ the Gaussian puff atmospheric dispersion model to forward simulate the transport of methane from the sources to the sensors. This model does not account for turbulence or the fact that large obstructions (e.g., buildings) can block the flow of methane, which would likely have a larger impact on the DLQ output on more complicated sites.

## Supporting Information Available

Additional details about the localization and quantification comparisons; Specifically, we tabulate the exact localization counts shown in Figure 3 and provide QQ plots for comparing concentration and emission rate distributions; Additionally, the SI contains further investigations into background removal, the effect of different temporal aggregation lengths, and the effect of conditioning the quantification comparison on identical localization estimates (PDF)

## References

[ref1] Alvarez R. A.; et al. Assessment of methane emissions from the U.S. oil and gas supply chain. Science 2018, 361, 186–188. 10.1126/science.aar7204. 29930092 PMC6223263

[ref2] Chan E.; Worthy D. E. J.; Chan D.; Ishizawa M.; Moran M. D.; Delcloo A.; Vogel F. Eight-Year Estimates of Methane Emissions from Oil and Gas Operations in Western Canada Are Nearly Twice Those Reported in Inventories. Environ. Sci. Technol. 2020, 54, 14899–14909. 10.1021/acs.est.0c04117. 33169990

[ref3] Rutherford J. S.; Sherwin E. D.; Ravikumar A. P.; Heath G. A.; Englander J.; Cooley D.; Lyon D.; Omara M.; Langfitt Q.; Brandt A. R. Closing the methane gap in US oil and natural gas production emissions inventories. Nat. Commun. 2021, 12, 4715 10.1038/s41467-021-25017-4. 34354066 PMC8342509

[ref4] Conrad B. M.; Tyner D. R.; Li H. Z.; Xie D.; Johnson M. R. A measurement-based upstream oil and gas methane inventory for Alberta, Canada reveals higher emissions and different sources than official estimates. Communications Earth & Environment 2023, 4, 1–10. 10.1038/s43247-023-01081-0.37325084

[ref5] Yarmuth J. A. H.R.5376 - 117th Congress (2021–2022): Inflation Reduction Act of 2022. https://www.congress.gov/bill/117th-congress/house-bill/5376 (accessed March 6, 2025).

[ref6] U.S. Environmental Protection Agency. Greenhouse Gas Reporting Rule: Revisions and Confidentiality Determinations for Petroleum and Natural Gas Systems; Final Rule 89 FR 42062, 2024; pp 42062–42327, https://www.federalregister.gov/d/2024-08988 (accessed March 6, 2025).

[ref7] Council of European Union. Council regulation (EU) 2019/942 (COM(2021)0805 — C9–0467/2021 — 2021/0423(COD)). 2023; https://www.europarl.europa.eu/doceo/document/TA-9-2023-0127_EN.html (accessed March 6, 2025).

[ref8] UN Environment Programme (UNEP). The Oil & Gas Methane Partnership 2.0 (OGMP 2.0). https://ogmpartnership.com/ (accessed March 6, 2025).

[ref9] Johnson M. R.; Tyner D. R.; Szekeres A. J. Blinded evaluation of airborne methane source detection using Bridger Photonics LiDAR. Remote Sensing of Environment 2021, 259, 112418 10.1016/j.rse.2021.112418.

[ref10] Sherwin E. D.; Chen Y.; Ravikumar A. P.; Brandt A. R. Single-blind test of airplane-based hyperspectral methane detection via controlled releases. Elementa: Science of the Anthropocene 2021, 9, 00063 10.1525/elementa.2021.00063.

[ref11] El Abbadi S. H.; Chen Z.; Burdeau P. M.; Rutherford J. S.; Chen Y.; Zhang Z.; Sherwin E. D.; Brandt A. R. Technological Maturity of Aircraft-Based Methane Sensing for Greenhouse Gas Mitigation. Environ. Sci. Technol. 2024, 58, 9591–9600. 10.1021/acs.est.4c02439. 38759639 PMC11154951

[ref12] Sherwin E. D.; El Abbadi S. H.; Burdeau P. M.; Zhang Z.; Chen Z.; Rutherford J. S.; Chen Y.; Brandt A. R. Single-blind test of nine methane-sensing satellite systems from three continents. Atmospheric Measurement Techniques 2024, 17, 765–782. 10.5194/amt-17-765-2024.

[ref13] Bell C.; Rutherford J.; Brandt A.; Sherwin E.; Vaughn T.; Zimmerle D. Single-blind determination of methane detection limits and quantification accuracy using aircraft-based LiDAR. Elementa: Science of the Anthropocene 2022, 10, 00080 10.1525/elementa.2022.00080.

[ref14] Conrad B. M.; Tyner D. R.; Johnson M. R. Robust probabilities of detection and quantification uncertainty for aerial methane detection: Examples for three airborne technologies. Remote Sensing of Environment 2023, 288, 113499 10.1016/j.rse.2023.113499.

[ref15] Schissel C.; Allen D. T. Impact of the High-Emission Event Duration and Sampling Frequency on the Uncertainty in Emission Estimates. Environmental Science & Technology Letters 2022, 9, 1063–1067. 10.1021/acs.estlett.2c00731.

[ref16] Daniels W. S.; Wang J. L.; Ravikumar A. P.; Harrison M.; Roman-White S. A.; George F. C.; Hammerling D. M. Toward Multiscale Measurement-Informed Methane Inventories: Reconciling Bottom-Up Site-Level Inventories with Top-Down Measurements Using Continuous Monitoring Systems. Environ. Sci. Technol. 2023, 57, 11823–11833. 10.1021/acs.est.3c01121. 37506319 PMC10433519

[ref17] Lavoie T. N.; Shepson P. B.; Cambaliza M. O. L.; Stirm B. H.; Conley S.; Mehrotra S.; Faloona I. C.; Lyon D. Spatiotemporal Variability of Methane Emissions at Oil and Natural Gas Operations in the Eagle Ford Basin. Environ. Sci. Technol. 2017, 51, 8001–8009. 10.1021/acs.est.7b00814. 28678487

[ref18] Schwietzke S.; et al. Improved Mechanistic Understanding of Natural Gas Methane Emissions from Spatially Resolved Aircraft Measurements. Environ. Sci. Technol. 2017, 51, 7286–7294. 10.1021/acs.est.7b01810. 28548824

[ref19] Vaughn T. L.; Bell C. S.; Pickering C. K.; Schwietzke S.; Heath G. A.; Pétron G.; Zimmerle D. J.; Schnell R. C.; Nummedal D. Temporal variability largely explains top-down/bottom-up difference in methane emission estimates from a natural gas production region. Proceedings of the National Academy of Sciencesn 2018, 115, 11712–11717. 10.1073/pnas.1805687115. PMC6243284 30373838

[ref20] Cusworth D. H.; Duren R. M.; Thorpe A. K.; Olson-Duvall W.; Heckler J.; Chapman J. W.; Eastwood M. L.; Helmlinger M. C.; Green R. O.; Asner G. P.; Dennison P. E.; Miller C. E. Intermittency of Large Methane Emitters in the Permian Basin. Environmental Science & Technology Letters 2021, 8, 567–573. 10.1021/acs.estlett.1c00173.

[ref21] Wang J. L.; Daniels W. S.; Hammerling D. M.; Harrison M.; Burmaster K.; George F. C.; Ravikumar A. P. Multiscale Methane Measurements at Oil and Gas Facilities Reveal Necessary Frameworks for Improved Emissions Accounting. Environ. Sci. Technol. 2022, 56, 14743–14752. 10.1021/acs.est.2c06211. 36201663 PMC9583612

[ref22] Chen Y.; Sherwin E. D.; Berman E. S.; Jones B. B.; Gordon M. P.; Wetherley E. B.; Kort E. A.; Brandt A. R. Quantifying Regional Methane Emissions in the New Mexico Permian Basin with a Comprehensive Aerial Survey. Environ. Sci. Technol. 2022, 56, 4317–4323. 10.1021/acs.est.1c06458. 35317555

[ref23] Sherwin E. D.; Rutherford J. S.; Zhang Z.; Chen Y.; Wetherley E. B.; Yakovlev P. V.; Berman E. S. F.; Jones B. B.; Cusworth D. H.; Thorpe A. K.; Ayasse A. K.; Duren R. M.; Brandt A. R. US oil and gas system emissions from nearly one million aerial site measurements. Nature 2024, 627, 328–334. 10.1038/s41586-024-07117-5. 38480966

[ref24] Tullos E. E.; Stokes S. N.; Cardoso-Saldaña F. J.; Herndon S. C.; Smith B. J.; Allen D. T. Use of Short Duration Measurements to Estimate Methane Emissions at Oil and Gas Production Sites. Environmental Science & Technology Letters 2021, 8, 463–467. 10.1021/acs.estlett.1c00239.

[ref25] Johnson M. R.; Conrad B. M.; Tyner D. R. Creating measurement-based oil and gas sector methane inventories using source-resolved aerial surveys. Communications Earth & Environment 2023, 4, 1–9. 10.1038/s43247-023-00769-7.37325084

[ref26] Ravikumar A. P.; et al. Measurement-based differentiation of low-emission global natural gas supply chains. Nature Energy 2023, 8, 1174–1176. 10.1038/s41560-023-01381-x.

[ref27] Allen D.; Ravikumar A.; Tullos E. Scientific Challenges of Monitoring, Measuring, Reporting, and Verifying Greenhouse Gas Emissions from Natural Gas Systems. ACS Sustainable Resource Management 2024, 1, 10–12. 10.1021/acssusresmgt.3c00132.

[ref28] Alden C. B.; Ghosh S.; Coburn S.; Sweeney C.; Karion A.; Wright R.; Coddington I.; Rieker G. B.; Prasad K. Bootstrap inversion technique for atmospheric trace gas source detection and quantification using long open-path laser measurements. Atmospheric Measurement Techniques 2018, 11, 1565–1582. 10.5194/amt-11-1565-2018.

[ref29] Cartwright L.; Zammit-Mangion A.; Bhatia S.; Schroder I.; Phillips F.; Coates T.; Negandhi K.; Naylor T.; Kennedy M.; Zegelin S.; Wokker N.; Deutscher N. M.; Feitz A. Bayesian atmospheric tomography for detection and quantification of methane emissions: application to data from the 2015 Ginninderra release experiment. Atmospheric Measurement Techniques 2019, 12, 4659–4676. 10.5194/amt-12-4659-2019.

[ref30] Kumar P.; et al. Near-field atmospheric inversions for the localization and quantification of controlled methane releases using stationary and mobile measurements. Quarterly Journal of the Royal Meteorological Society 2022, 148, 1886–1912. 10.1002/qj.4283.

[ref31] Daniels W. S.; Jia M.; Hammerling D. M. Detection, localization, and quantification of single-source methane emissions on oil and gas production sites using point-in-space continuous monitoring systems. Elementa: Science of the Anthropocene 2024, 12, 00110 10.1525/elementa.2023.00110.

[ref32] Daniels W. S.; Jia M.; Hammerling D. M. Estimating Methane Emission Durations Using Continuous Monitoring Systems. Environmental Science & Technology Letters 2024, 11, 1187–1192. 10.1021/acs.estlett.4c00687. 39554602 PMC11562728

[ref33] Chen Q.; Schissel C.; Kimura Y.; McGaughey G.; McDonald-Buller E.; Allen D. T. Assessing Detection Efficiencies for Continuous Methane Emission Monitoring Systems at Oil and Gas Production Sites. Environ. Sci. Technol. 2023, 57, 1788–1796. 10.1021/acs.est.2c06990. 36652306

[ref34] Chen Q.; Kimura Y.; Allen D. Determining times to detection for large methane release events using continuously operating methane sensing systems at simulated oil and gas production sites. 2023, v1. ChemRxiv. https://doi.org/10.26434/chemrxiv-2023-p8lfk (accessed March 6, 2025).

[ref35] Bell C. S.; Vaughn T.; Zimmerle D. Evaluation of next generation emission measurement technologies under repeatable test protocols. Elementa: Science of the Anthropocene 2020, 8, 32 10.1525/elementa.426.

[ref36] Bell C.; Ilonze C.; Duggan A.; Zimmerle D. Performance of Continuous Emission Monitoring Solutions under a Single-Blind Controlled Testing Protocol. Environ. Sci. Technol. 2023, 57, 5794–5805. 10.1021/acs.est.2c09235. 36977200 PMC10100557

[ref37] Ilonze C.; Emerson E.; Duggan A.; Zimmerle D. Assessing the Progress of the Performance of Continuous Monitoring Solutions under a Single-Blind Controlled Testing Protocol. Environ. Sci. Technol. 2024, 58, 10941–10955. 10.1021/acs.est.3c08511. 38865299 PMC11210203

[ref38] Chen Z.; El Abbadi S. H.; Sherwin E. D.; Burdeau P. M.; Rutherford J. S.; Chen Y.; Zhang Z.; Brandt A. R. Comparing Continuous Methane Monitoring Technologies for High-Volume Emissions: A Single-Blind Controlled Release Study. ACS ES&T Air 2024, 1, 871–884. 10.1021/acsestair.4c00015.

[ref39] Cheptonui F.; Emerson E.; Ilonze C.; Day R., Levin E.; Fleischmann D.; Brouwer R.; Zimmerle D. Assessing the Performance of Emerging and Existing Continuous Monitoring Solutions under a Single-blind Controlled Testing Protocol. 2024, v1. ChemRxiv. https://chemrxiv.org/engage/chemrxiv/article-details/673f839a5a82cea2fa46b483 (accessed March 6, 2025).

[ref40] Day R. E.; Emerson E.; Bell C.; Zimmerle D. Point Sensor Networks Struggle to Detect and Quantify Short Controlled Releases at Oil and Gas Sites. Sensors 2024, 24, 2419 10.3390/s24082419. 38676036 PMC11054334

[ref41] Yang S. L.; Ravikumar A. P. Assessing the Performance of Point Sensor Continuous Monitoring Systems at Midstream Natural Gas Compressor Stations. ACS EST Air 2025, 10.1021/acsestair.4c00227.

[ref42] Thorpe M. J.; et al. Deployment-invariant probability of detection characterization for aerial LiDAR methane detection. Remote Sensing of Environment 2024, 315, 114435 10.1016/j.rse.2024.114435.

[ref43] Jia M.; Sorensen T. R.; Hammerling D. M. Optimizing Point-in-Space Continuous Monitoring System Sensor Placement on Oil and Gas Sites. ACS Sustainable Resource Management 2025, 2, 72–81. 10.1021/acssusresmgt.4c00333. 39877196 PMC11770763

[ref44] Jia M.; Fish R.; Daniels W. S.; Sprinkle B.; Hammerling D. M. Filling a critical need: a lightweight and fast Gaussian puff model implementation. 2024, v3. ChemRxiv. https://chemrxiv.org/engage/chemrxiv/article-details/672a296b7be152b1d00fcc60 (accessed March 6, 2025). 10.1038/s41598-025-99491-x PMC12119887 40437081

[ref45] Pennsylvania Department of Environmental Protection. https://www.pa.gov/agencies/dep.html (accessed March 6, 2025).

[ref46] United States Environmental Protection Agency (EPA). Greenhouse Gas Reporting Program (GHGRP) Subpart W – Petroleum and Natural Gas Systems. https://www.epa.gov/ghgreporting/subpart-w-petroleum-and-natural-gas-systems (accessed March 6, 2025).

[ref47] Goetz J. D.; Avery A.; Werden B.; Floerchinger C.; Fortner E. C.; Wormhoudt J.; Massoli P.; Herndon S. C.; Kolb C. E.; Knighton W. B.; Peischl J.; Warneke C.; de Gouw J. A.; Shaw S. L.; DeCarlo P. F. Analysis of local-scale background concentrations of methane and other gas-phase species in the Marcellus Shale. Elementa: Science of the Anthropocene 2017, 5, 1 10.1525/elementa.182.

[ref48] United States Environmental Protection Agency (EPA). Standards of Performance for New, Reconstructed, and Modified Sources and Emissions Guidelines for Existing Sources: Oil and Natural Gas Sector Climate Review, 40 CFR Part 60. https://www.federalregister.gov/d/2024-00366 (accessed March 6, 2025).

