# Supplementary material for: Intercomparison of Three Continuous Monitoring Systems on Operating Oil and Gas Sites
Source: ACS EST Air. 2025 Mar 18;2(4):564–77. doi: 10.1021/acsestair.4c00298 (PMC11997984; doi:10.1021/acsestair.4c00298)
Supplement: Supplementary file 1 — ea4c00298_si_001.pdf [file ea4c00298_si_001.pdf]

Supporting Information for:

# Intercomparison of three continuous monitoring systems on operating oil and gas sites

William S. Daniels[†,1], Spencer G. Kidd[†,1], Shuting (Lydia) Yang[2], Shannon Stokes[3], Arvind P. Ravikumar[2,4], and Dorit M. Hammerling[1,4]

[1]Department of Applied Mathematics and Statistics, Colorado School of Mines, Golden, Colorado 80401, United States

[2]Department of Petroleum and Geosystems Engineering, The University of Texas at Austin, Austin, Texas 78712, United States

[3]Center for Energy and Environmental Resources, The University of Texas at Austin, Austin, Texas 78712, United States

[4]Energy Emissions Modeling and Data Lab, The University of Texas at Austin, Austin, Texas 78712, United States

† denotes equal contribution

Email: wdaniels@mines.edu

## Contents

**S1 Concentration data without upsampling Solution B measurements** 2

**S2 Background removed concentration data** 4

**S3 Full localization results** 5

**S4 Near real time parity plots with confidence intervals** 12

**S5 QQ plots for concentration measurements and estimated emission rates** 13

**S6 Temporal aggregation effects on quantification data** 14

**S7 Quantification comparison conditioned on the same localization estimate** 16

## S1 Concentration data without upsampling Solution B measurements

Figure S1 shows the same concentration comparison as in Figure 2 in the main manuscript, but uses the native data product from Solution B without any upsampling. As discussed in the main manuscript, Solution B only reports a new concentration value when a notable change is detected, and as such, their native data product looks much sparser than that of the other CMS solutions.

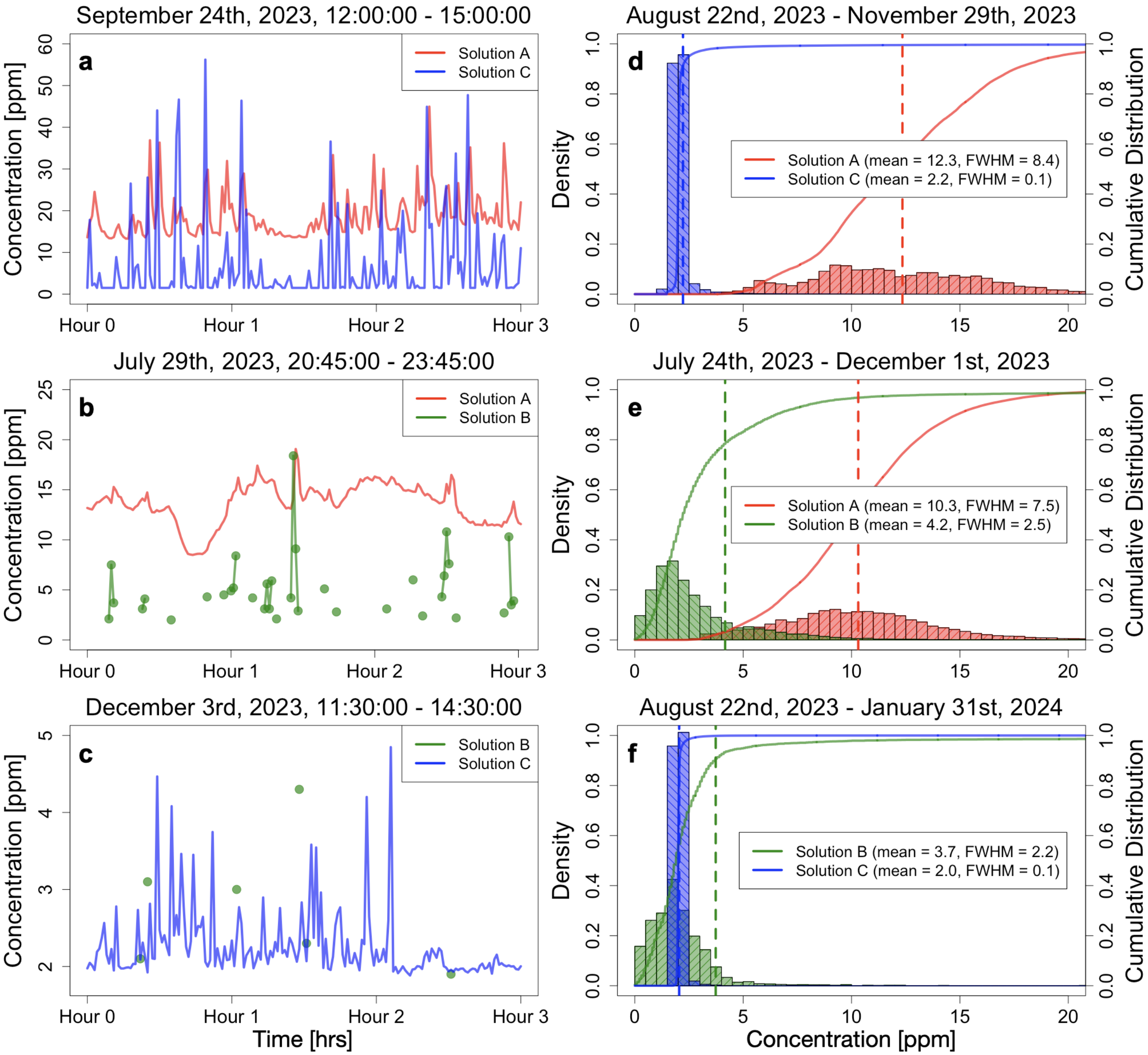


Figure S1: Concentration data from the nearly co-located sensor pairs, with each row showing data from a different sensor pair. Solution B measurements have not been upsampled. See Figure 1 in the main manuscript for the location of each sensor pair shown here. (a)-(c) zoom in on a representative three hour period to show detail. Note that (a)-(c) have different vertical scales, as they show data from different sites and time periods and hence are not meant to be directly compared. (d)-(f) show the distribution of concentration measurements from the entire time period during which both solutions were deployed. Solid lines show the empirical cumulative distribution functions, and vertical dashed lines show the distribution average. Full width half maximum (FWHM) values are listed for each solution.

The distribution of the native data product from Solution B has a heavier tail and a higher mean than the upsampled version shown in Figure 2 of the main text. This can be seen in subfigures (e) and (f), where the mean of the Solution B measurements is now 4.2 and 3.7 ppm, respectively, compared to 2.0 and 1.9 ppm in Figure 2 of the main text. This is because periods of elevated methane concentrations are less frequent than periods of background concentrations, but contain more rapidly changing concentration values. In other words, periods of background methane concentrations on the oil and gas sites remain fairly constant around the regional background of approximately 2.0 ppm, while periods of elevated methane concentrations rise and decline rapidly. As such, few background observations are reported by Solution B, since they rarely exhibit a "notable change." However, almost all of the enhancements are reported, as these change rapidly. Therefore, the Solution B concentration distributions in subfigure (e) and (f) are artificially skewed to the right. For this reason, we believe the upsampling procedure used in the main manuscript is a better representation of Solution B's concentration data. This is supported by the fact that the mean of the upsampled Solution B concentration data in Figure 2 of the main text is very close to the regional background, as expected. The mean of the native data product shown in Figure S1 is much higher than the regional background, which is not expected, since a majority of the concentration measurements on oil and gas sites should be close to the regional background.

We expect the upsampling procedure described in the main text to have minimal impact on the DLQ results when using Solution B's data. This is because repeating the native Solution B data until a new measurement is reported is the closest approximation of the true underlying methane signal. The fact that the upsampled data has no variability will not impact the DLQ algorithms. However, the fidelity of the resulting DLQ output will depend on the quality of the change detection algorithm used by Solution B to report data. For example, if Solution B's algorithm does not flag a concentration enhancement as a "notable change," then that enhancement would be missed and the resulting quantification estimate for that time period would be underestimated. Since we do not have access to the original Solution B data (before they perform their "notable change" filtering), we cannot assess the fidelity of this process.

## S2 Background removed concentration data

Figure S2 shows the same concentration data as Figure 2 in the main manuscript, but background-corrected using the DLQ algorithm. This background-correction procedure is described in the main manuscript, and additional details can be found in [1].

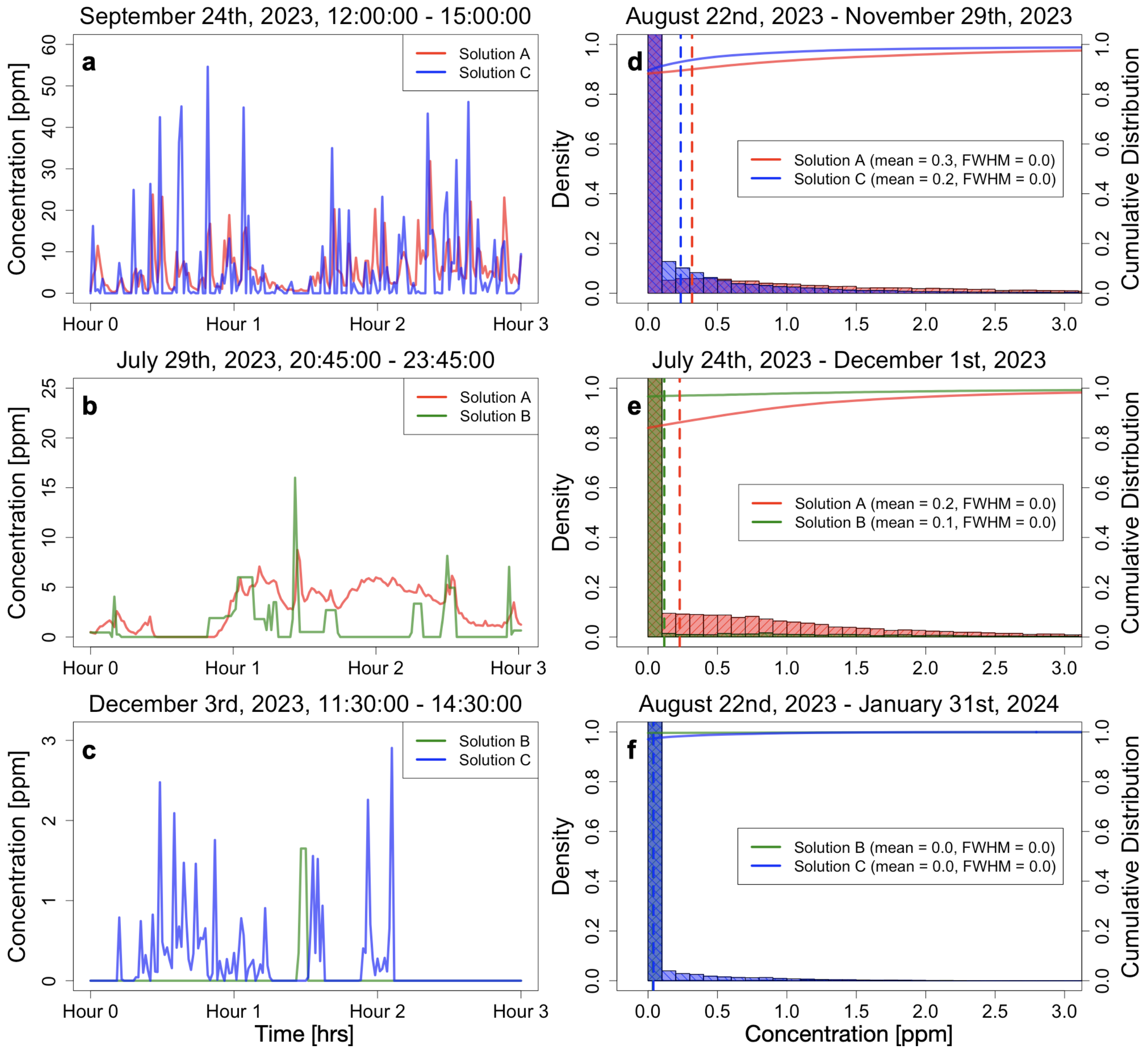


Figure S2: Background-corrected concentration data from the nearly co-located sensor pairs, with each row showing data from a different sensor pair. See Figure 1 in the main manuscript for the location of each sensor pair shown here. (a)-(c) zoom in on a representative three hour period to show detail. Note that (a)-(c) have different vertical scales, as they show data from different sites and time periods and hence are not meant to be directly compared. (d)-(f) show the distribution of concentration measurements from the entire time period during which both solutions were deployed. Solid lines show the empirical cumulative distribution functions, and vertical dashed lines show the distribution average. Full width half maximum (FWHM) values are listed for each solution.

## S3 Full localization results

Tables S1 through S6 show data from Figure 3 in the main text in more detail. Each Table shows the exact number of localization estimates per equipment group for a given site. For each equipment group there are 3 rows: "Non-alignment", "Alignment", and "Source total". "Non-alignment" lists the number of 30-minute intervals that each solution localized to a given equipment group when the other solution did not localize to that equipment group. This number will not necessarily be the same between solutions. "Alignment" lists the number of 30-minute intervals that both solutions localized to a given equipment group at the same time. This number, by definition, will be the same between solutions. "Source total" lists the total number of 30-minute intervals that each solution localized to a given equipment group. Finally, "Site total" lists the total number of 30-minute intervals during the deployment period. This number will be the same between solutions because we only use the time period when both solutions were deployed on a given site.

Consider Table S1 as an example. For this site, there were 391 intervals where Solution A localized to the Separator and Solution C localized to a different equipment group. There were 331 intervals where Solution C localized to the Separator and Solution A localized to a different equipment group. There were 26 intervals where both solutions localized to the Separator.

In Table 3 of the main manuscript we define two localization metrics: "Percent of estimates that align in time" and "Percent of estimates that align in distribution". For the site in Table S1, these values are 23.1% and 83.6% respectively. "Percent of estimates that align in time" is calculated by summing up all the "Aligned" values and dividing by the "Site total" value:

$$(26 + 11 + 119 + 53 + 56 + 64 + 1031)/5893 \approx 0.231.$$

"Percent of estimates that align in distribution" quantifies how similar the localization estimates are in distribution, regardless of if they were made at the same time between solutions. This is calculated by summing up all the "Aligned" values as well as the lower of the two "Non-aligned" values and dividing by the "Site total" value:

$$[(26 + 11 + 119 + 53 + 56 + 64 + 1031) + (331 + 185 + 554 + 542 + 420 + 402 + 1133)]/5893 \approx 0.836.$$

Table S1: Localization estimates for Site 1. Rows show the potential emission sources on the site. The "Non-aligned" row lists the number of intervals where a solution localized to a given source and the other solution localized to a different source. The "Aligned" row lists the number of intervals where both solutions localize to the same source. The "Source total" row lists the total number of intervals that each solution localized to a given source. "Site total" lists the total number of intervals during the deployment period.

| **Site 1** | | Solution A | Solution C |
|---|---|---|---|
| Separator | Non-aligned | 391 | 331 |
| | Aligned | 26 | |
| | Source total | 417 | 357 |
| VDU | Non-aligned | 273 | 185 |
| | Aligned | 11 | |
| | Source total | 284 | 196 |
| Tank | Non-aligned | 554 | 711 |
| | Aligned | 119 | |
| | Source total | 673 | 830 |
| Wellheads | Non-aligned | 542 | 880 |
| | Aligned | 53 | |
| | Source total | 595 | 933 |
| Dehydrator | Non-aligned | 420 | 881 |
| | Aligned | 56 | |
| | Source total | 476 | 937 |
| Compressors | Non-aligned | 1220 | 402 |
| | Aligned | 64 | |
| | Source total | 1284 | 466 |
| No Emissions | Non-aligned | 1133 | 1143 |
| | Aligned | 1031 | |
| | Source total | 2164 | 2174 |
| **Site total** | | 5893 | |

Table S2: Localization estimates for Site 2. Rows show the potential emission sources on the site. The "Non-aligned" row lists the number of intervals where a solution localized to a given source and the other solution localized to a different source. The "Aligned" row lists the number of intervals where both solutions localize to the same source. The "Source total" row lists the total number of intervals that each solution localized to a given source. "Site total" lists the total number of intervals during the deployment period.

| **Site 2** | | Solution A | Solution C |
|---|---|---|---|
| Wellheads | Non-aligned | 424 | 676 |
| | Aligned | 30 | |
| | Source total | 454 | 706 |
| Compressors | Non-aligned | 615 | 257 |
| | Aligned | 59 | |
| | Source total | 674 | 316 |
| Flash Vessel | Non-aligned | 1006 | 255 |
| | Aligned | 41 | |
| | Source total | 1047 | 296 |
| GPU | Non-aligned | 1281 | 222 |
| | Aligned | 76 | |
| | Source total | 1357 | 298 |
| Dehydrator | Non-aligned | 1410 | 122 |
| | Aligned | 108 | |
| | Source total | 1518 | 230 |
| No Emissions | Non-aligned | 307 | 3511 |
| | Aligned | 535 | |
| | Source total | 842 | 4046 |
| **Site total** | | 5892 | |

Table S3: Localization estimates for Site 3. Rows show the potential emission sources on the site. The "Non-aligned" row lists the number of intervals where a solution localized to a given source and the other solution localized to a different source. The "Aligned" row lists the number of intervals where both solutions localize to the same source. The "Source total" row lists the total number of intervals that each solution localized to a given source. "Site total" lists the total number of intervals during the deployment period.

| **Site 3** | | Solution A | Solution B |
|---|---|---|---|
| Separator | Non-aligned | 187 | 84 |
| | Aligned | 0 | |
| | Source total | 187 | 84 |
| Tank | Non-aligned | 528 | 399 |
| | Aligned | 0 | |
| | Source total | 528 | 399 |
| Flare | Non-aligned | 650 | 394 |
| | Aligned | 81 | |
| | Source total | 731 | 475 |
| Wellheads | Non-aligned | 589 | 249 |
| | Aligned | 38 | |
| | Source total | 627 | 287 |
| GPU | Non-aligned | 634 | 224 |
| | Aligned | 12 | |
| | Source total | 646 | 236 |
| Compressors | Non-aligned | 2153 | 808 |
| | Aligned | 223 | |
| | Source total | 2376 | 1031 |
| No Emissions | Non-aligned | 207 | 2790 |
| | Aligned | 955 | |
| | Source total | 1162 | 3745 |
| **Site total** | | 6257 | |

Table S4: Localization estimates for Site 4. Rows show the potential emission sources on the site. The "Non-aligned" row lists the number of intervals where a solution localized to a given source and the other solution localized to a different source. The "Aligned" row lists the number of intervals where both solutions localize to the same source. The "Source total" row lists the total number of intervals that each solution localized to a given source. "Site total" lists the total number of intervals during the deployment period.

| **Site 4** | | Solution A | Solution B |
|---|---|---|---|
| Gas Scrubber | Non-aligned | 1186 | 1076 |
| | Aligned | 783 | |
| | Source total | 1969 | 1859 |
| Pump | Non-aligned | 794 | 678 |
| | Aligned | 164 | |
| | Source total | 958 | 842 |
| GPU | Non-aligned | 413 | 635 |
| | Aligned | 72 | |
| | Source total | 485 | 707 |
| Wellheads | Non-aligned | 309 | 1364 |
| | Aligned | 169 | |
| | Source total | 478 | 1533 |
| Tank | Non-aligned | 2234 | 1105 |
| | Aligned | 0 | |
| | Source total | 2234 | 1105 |
| No Emissions | Non-aligned | 110 | 188 |
| | Aligned | 5 | |
| | Source total | 115 | 193 |
| **Site total** | | 6257 | |

Table S5: Localization estimates for Site 5. Rows show the potential emission sources on the site. The "Non-aligned" row lists the number of intervals where a solution localized to a given source and the other solution localized to a different source. The "Aligned" row lists the number of intervals where both solutions localize to the same source. The "Source total" row lists the total number of intervals that each solution localized to a given source. "Site total" lists the total number of intervals during the deployment period.

| **Site 5** | | Solution B | Solution C |
|---|---|---|---|
| Gas Scrubber | Non-aligned | 135 | 81 |
| | Aligned | 1 | |
| | Source total | 136 | 82 |
| Separator | Non-aligned | 137 | 31 |
| | Aligned | 0 | |
| | Source total | 137 | 31 |
| Dehydrator | Non-aligned | 180 | 324 |
| | Aligned | 3 | |
| | Source total | 183 | 327 |
| Compressors | Non-aligned | 874 | 715 |
| | Aligned | 61 | |
| | Source total | 935 | 776 |
| Control Room | Non-aligned | 249 | 74 |
| | Aligned | 0 | |
| | Source total | 249 | 74 |
| Tank | Non-aligned | 256 | 54 |
| | Aligned | 1 | |
| | Source total | 257 | 55 |
| Air Tank | Non-aligned | 246 | 41 |
| | Aligned | 4 | |
| | Source total | 250 | 45 |
| Flare | Non-aligned | 169 | 635 |
| | Aligned | 8 | |
| | Source total | 177 | 643 |
| Pipeline Header | Non-aligned | 641 | 4380 |
| | Aligned | 233 | |
| | Source total | 874 | 4613 |
| No Emissions | Non-aligned | 4111 | 663 |
| | Aligned | 2099 | |
| | Source total | 6210 | 2762 |
| **Site total** | | 9408 | |

Table S6: Localization estimates for Site 6. Rows show the potential emission sources on the site. The "Non-aligned" row lists the number of intervals where a solution localized to a given source and the other solution localized to a different source. The "Aligned" row lists the number of intervals where both solutions localize to the same source. The "Source total" row lists the total number of intervals that each solution localized to a given source. "Site total" lists the total number of intervals during the deployment period.

| **Site 6** | | Solution B | Solution C |
|---|---|---|---|
| Tank | Non-aligned | 490 | 576 |
| | Aligned | 54 | |
| | Source total | 544 | 630 |
| GPU | Non-aligned | 370 | 478 |
| | Aligned | 31 | |
| | Source total | 401 | 509 |
| BTEX Tank | Non-aligned | 684 | 563 |
| | Aligned | 47 | |
| | Source total | 731 | 610 |
| Heater | Non-aligned | 1222 | 1033 |
| | Aligned | 121 | |
| | Source total | 1343 | 1154 |
| VDU | Non-aligned | 143 | 630 |
| | Aligned | 21 | |
| | Source total | 164 | 651 |
| Gas Scrubber | Non-aligned | 761 | 160 |
| | Aligned | 22 | |
| | Source total | 783 | 182 |
| Wellheads | Non-aligned | 647 | 1945 |
| | Aligned | 104 | |
| | Source total | 751 | 2049 |
| Gas Header | Non-aligned | 2181 | 837 |
| | Aligned | 130 | |
| | Source total | 2311 | 967 |
| No Emissions | Non-aligned | 1548 | 1824 |
| | Aligned | 832 | |
| | Source total | 2380 | 2656 |
| **Site total** | | 5892 | |

# S4 Near real time parity plots with confidence intervals

Figure S3 shows the same parity plots as in Figure 4 in the main manuscript, but with 95% confidence intervals on the DLQ emission rate estimates. No confidence intervals are plotted for the vendor-provided rate estimates, as the CMS solution vendors did not provide any uncertainty quantification along with their emission rate estimates.

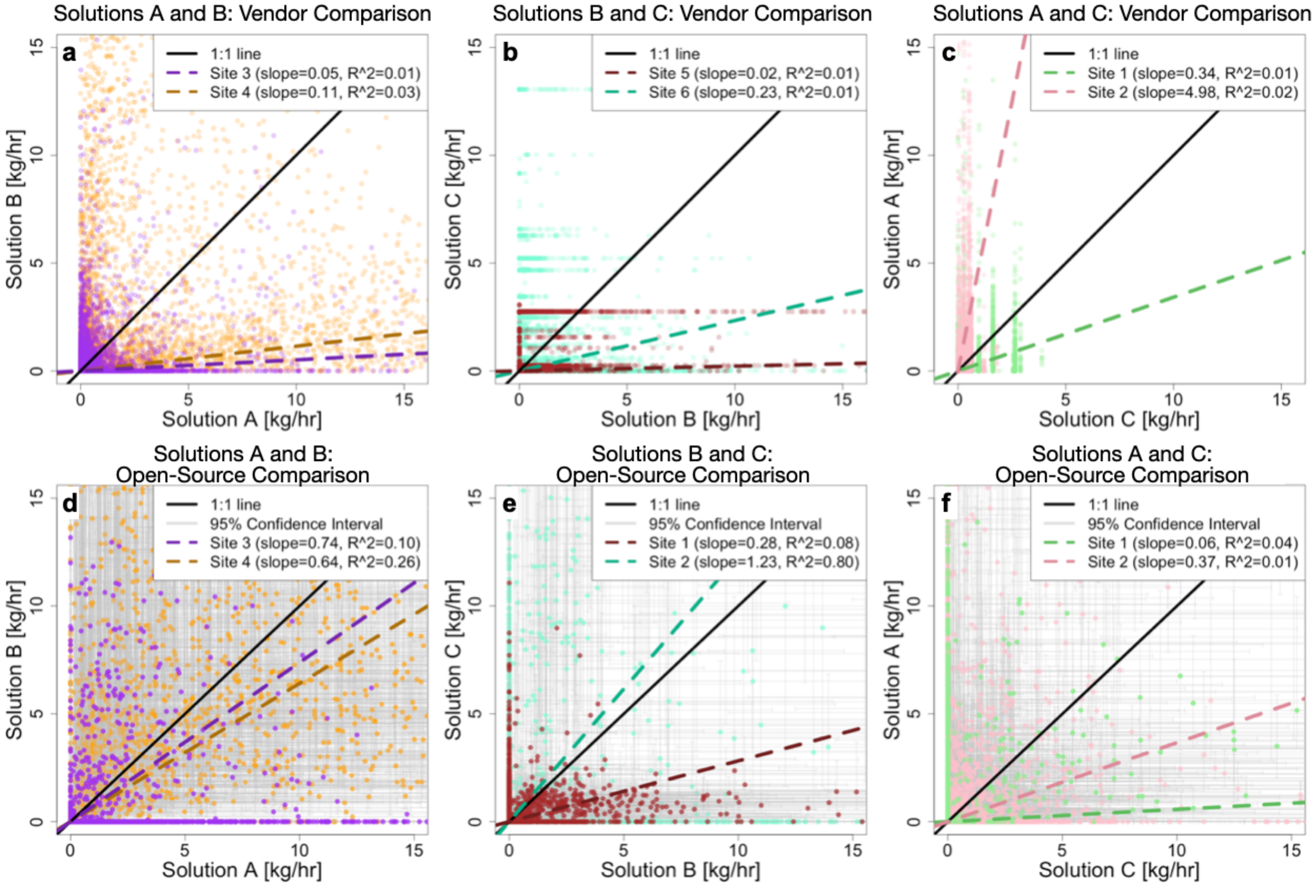


Figure S3: Parity plots comparing emission rate estimates made at the same time by the different CMS solutions. (a)-(c) compare rate estimates provided by the CMS vendors, and (d)-(f) compare rate estimates from the open-source DLQ algorithm applied to the raw concentration data from each CMS solution. Each point shows two rate estimates produced during one 30-minute quantification interval. Each subfigure uses data from the two oil and gas sites that have the two solutions installed. Axes are restricted to [0, 15] kg/hr to show detail.

## S5 QQ plots for concentration measurements and estimated emission rates

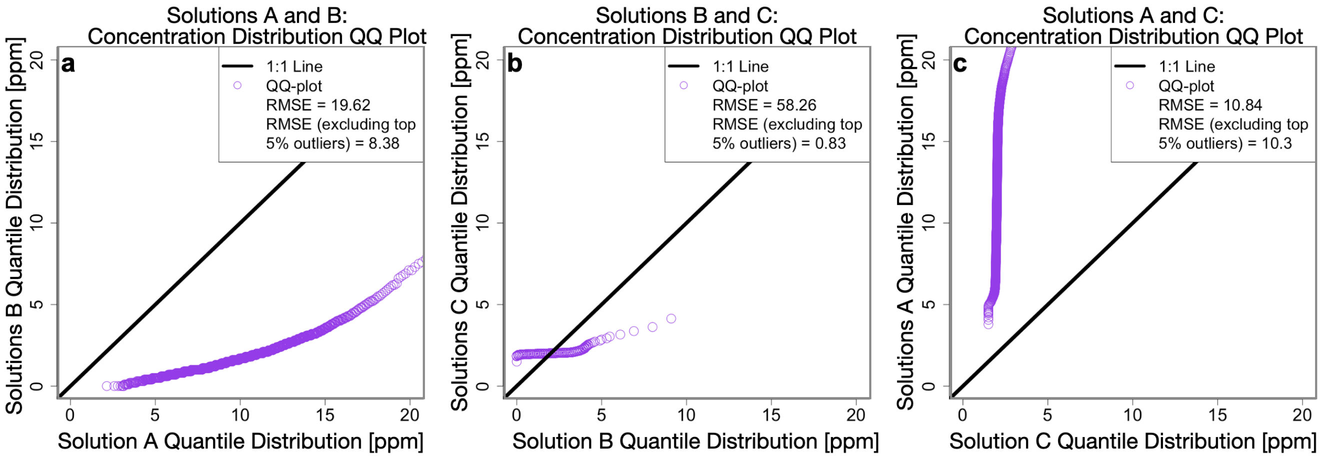


Figure S4: Quantile-quantile plots of the concentration measurements shown in Figure 2 of the main manuscript. The overall RMSE and the RMSE calculated after excluding the top 5% of the concentration observations is listed in the legend.

Figure S4 compares the distribution of concentration observations from Figure 2 in the main manuscript using quantile-quantile (QQ) plots. The root mean squared error (RMSE) for each comparison is shown in the top right of each subfigure. The RMSE is sensitive to outliers, so we also list the RMSE calculated after excluding the top 5% of the concentration observations to capture the bulk of the distribution. The axes are limited to [0, 20] ppm to show detail and align with Figure 2 in the main manuscript.

As in Figure 2 in the main manuscript, the distribution of Solution A is shifted higher than Solution B and C. Solution B and C have similar means, but Solution B is much heavier tailed compared to Solution C.

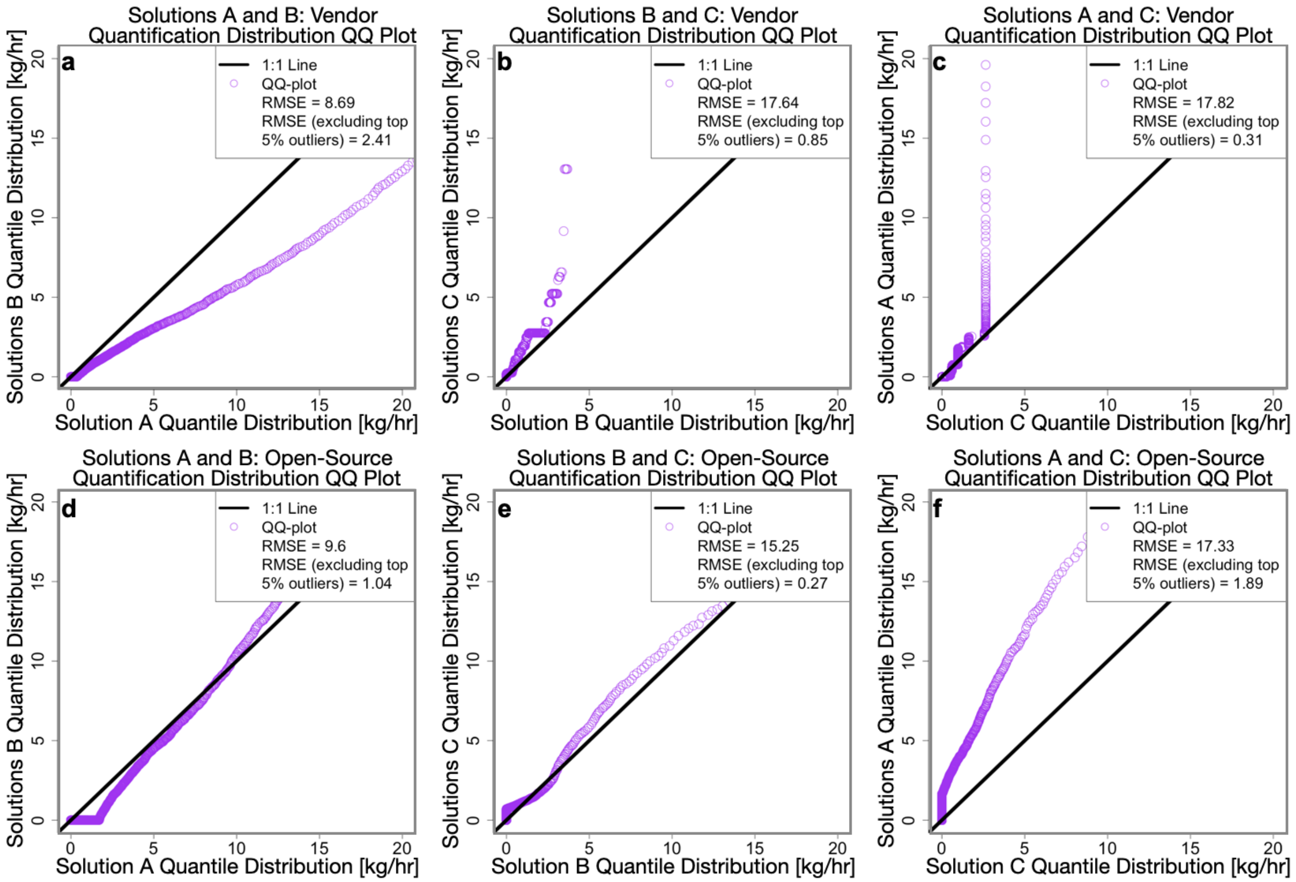


Figure S5: Quantile-quantile plots of the emission rate estimates shown in Figure 5 of the main manuscript. The overall RMSE and the RMSE calculated after excluding the top 5% of the emission rate estimates is listed in the legend.

Figure S5 shows the distribution of emission rate estimates from Figure 5 in the main manuscript. The root mean squared error (RMSE) for each comparison is shown in the top right of each subfigure. The RMSE is sensitive to outliers, so we also list the RMSE calculated after excluding the top 5% of the emission rate estimates to capture the bulk of the distribution. Subfigures (a)-(c) show QQ plots for the vendor-provided rate estimates, and subfigures (d)-(f) show QQ plots for the open-source rate estimates. The axes are limited to [0, 20] kg/hr to show detail.

As in Figure 5 in the main manuscript, the distribution of emission rate estimates are more similar in the bottom row when controlling for the quantification algorithm. This is much more prevalent for the Solution A-B and Solution B-C comparisons, as in Figure 5 in the main manuscript.

## S6 Temporal aggregation effects on quantification data

To better understand the improved agreement between quantification estimates at longer time scales, we calculate average emission rates using a range of temporal aggregation periods. This will demonstrate how temporally aggregated emission rates converge to the average taken across the entire time series. We consider temporal aggregations of 1 hour, 1 day, 1 week, 2 weeks, 1 month (30 days), and 2 months (60 days). These values were chosen to provide a range of aggregation periods between 30-minutes (the near real time analysis in the main manuscript) and the length of the entire time series (the distributional analysis in the main manuscript). For each aggregation period, we create non-overlapping intervals whose durations correspond to the length of the aggregation period. We then average the emission rate estimates within each interval.

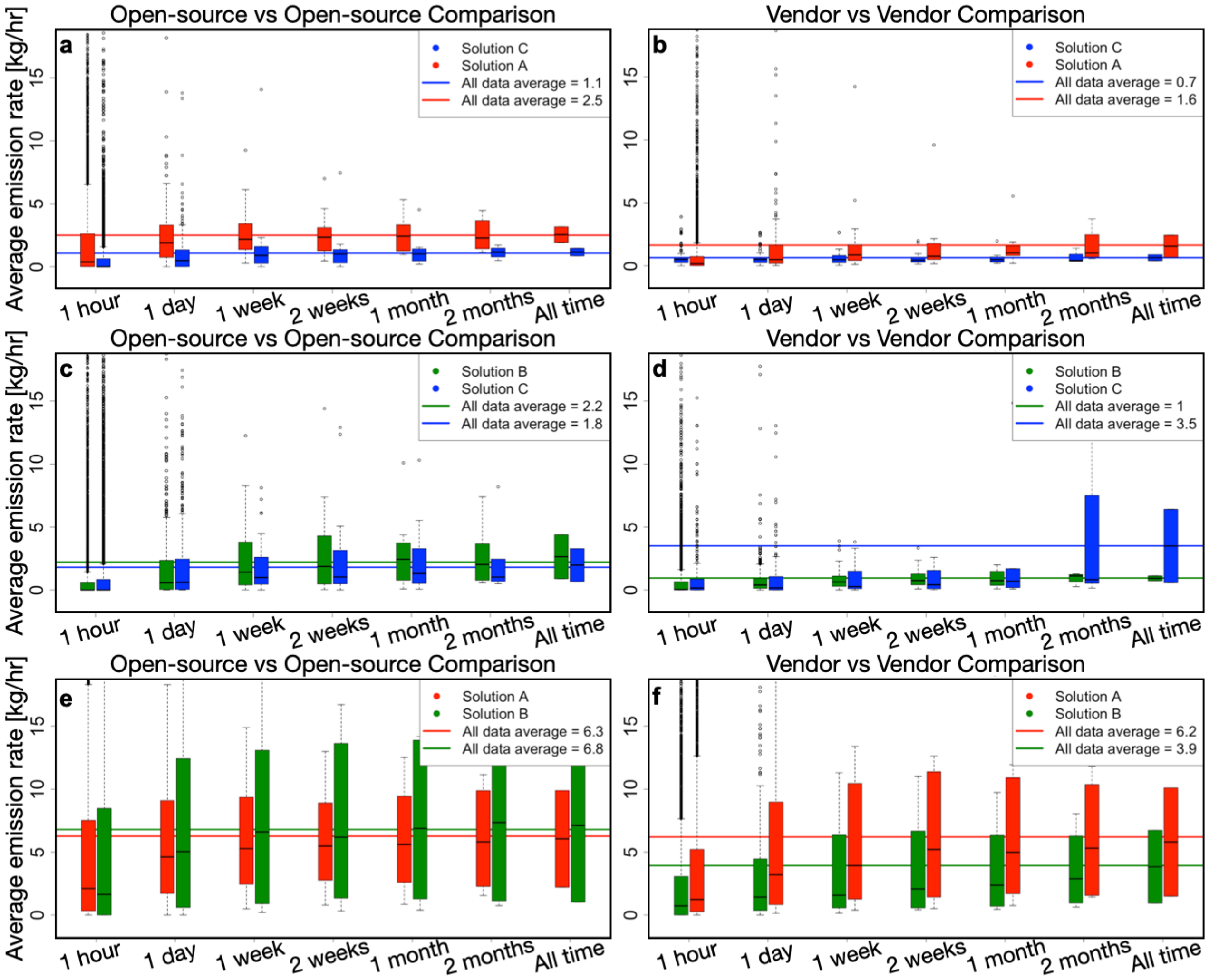


Figure S6: Distribution of mean quantification estimates at different temporal resolutions. Each row corresponds to a different pairwise comparison. Quantification estimates from the open-source DLQ algorithm are shown in the left column, and estimates from the vendors are in the right column. Each subfigure uses data from the two sites that have the two solutions installed (see Table 2 from the main manuscript). Vertical axes are restricted to [0, 15] kg/hr to show detail.

Figure S6 shows both open-source (left column) and vendor-provided (right column) quantification estimates averaged at the different temporal aggregation periods. The averages are shown as boxplots. There are more averages for the shorter temporal aggregation periods, as these periods have more non-overlapping intervals that fit within the entire time series. The horizontal lines show the average emission rate estimates across the entire time series. Note that 'All time' boxplots are generated using two all time averages (one for each site associated with the solution combination). These two 'All time' averages are generated by taking the average value of emission rate estimates during the entire deployment period of the solutions on each site. These deployment periods between sites are not always equivalent, meaning that the average between the two 'All time' mean values (i.e., the horizontal black line within the boxplots) will not always be the same as the mean value of all quantification estimates from those two sites (i.e., the colored horizontal lines spanning the width of the figure). This is simply due to one site having more or less available data than the other.

As expected based on the near real time analysis in the main manuscript, there is high variability in the average emission rates at short temporal aggregation periods. As the length of the temporal aggregation period increases, two primary things occur. First, the average of the emission rate averages (shown as the black line in the boxplots)

converges to the average emission rate across the entire time series. This is because averages over longer time periods will be closer to the average over the entire time series. Second, and more importantly, the variability in the emission rate averages decreases as the temporal aggregation period gets larger. Less variability in the emission rate averages means that there is more agreement between the two CMS solutions. Interestingly, most of the convergence occurs between the 1 day and 1 week aggregation periods. This means that CMS emission rate estimates averaged over only a few days will have similar agreement characteristics as the distributions shown in Figure 5 of the main manuscript.

## S7 Quantification comparison conditioned on the same localization estimate

Figure S7 compares near real time quantification estimates, similar to Figure 4 in the main manuscript, but only includes emission rate estimates where the corresponding localization estimate was the same between CMS solutions. This controls for another source of variability between the CMS solutions, as having a different localization estimate can impact the quantification estimate.

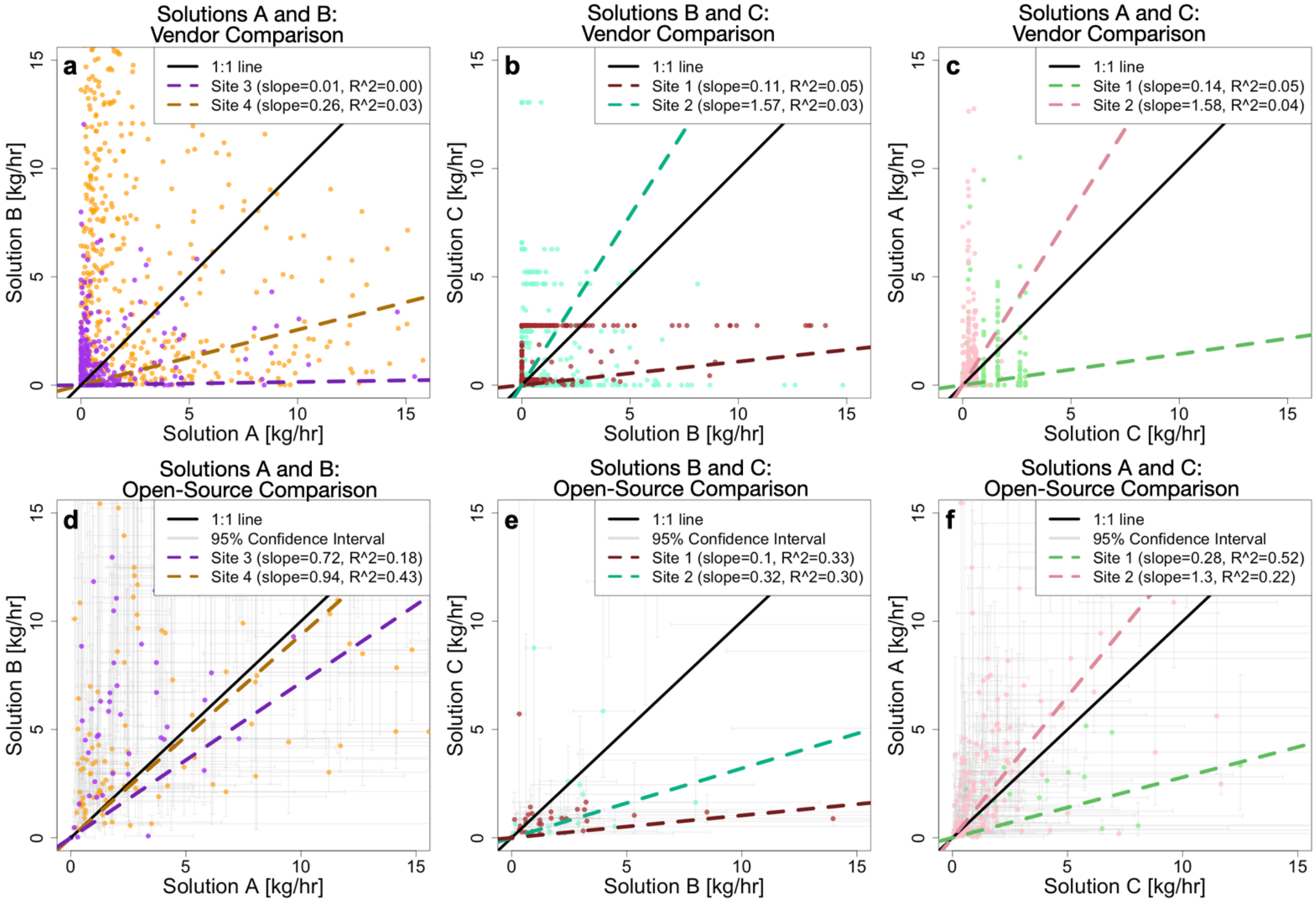


Figure S7: Parity plots comparing emission rate estimates made at the same time by the different CMS solutions, but only including the quantification estimates where the corresponding localization estimate was the same between CMS solutions. (a)-(c) compare rate estimates provided by the CMS vendors, and (d)-(f) compare rate estimates from the open-source DLQ algorithm applied to the raw concentration data from each CMS solution. Each point shows two rate estimates produced during one 30-minute quantification interval with (d)-(f) including a 95% confidence interval for each rate estimate. Each subfigure uses data from the two oil and gas sites that have the two solutions installed. Axes are restricted to [0, 15] kg/hr to show detail.

We still see generally poor alignment between solutions at the 30-minute scale even after controlling for the impact of different localization estimates. Best fit slopes range from 0.01 to 1.58 and $R^2$ values range from 0.00 to 0.52. Compared to Figure 4 in the main manuscript, the alignment between some solutions improved, such as the A-B comparison, while the alignment between others got worse, such as the B-C comparison. Whether or not the alignment improved after controlling for different localization estimates may be a function of the different site geometries or the number of remaining emission rate estimates.

Figure S8 compares emission rate estimates between the CMS solutions in distribution, similar to Figure 5 in the main manuscript, but only includes emission rate estimates where the corresponding localization estimates agreed between CMS solutions. This controls for another source of variability between the CMS solutions, as having a different localization estimate can impact the quantification estimate.

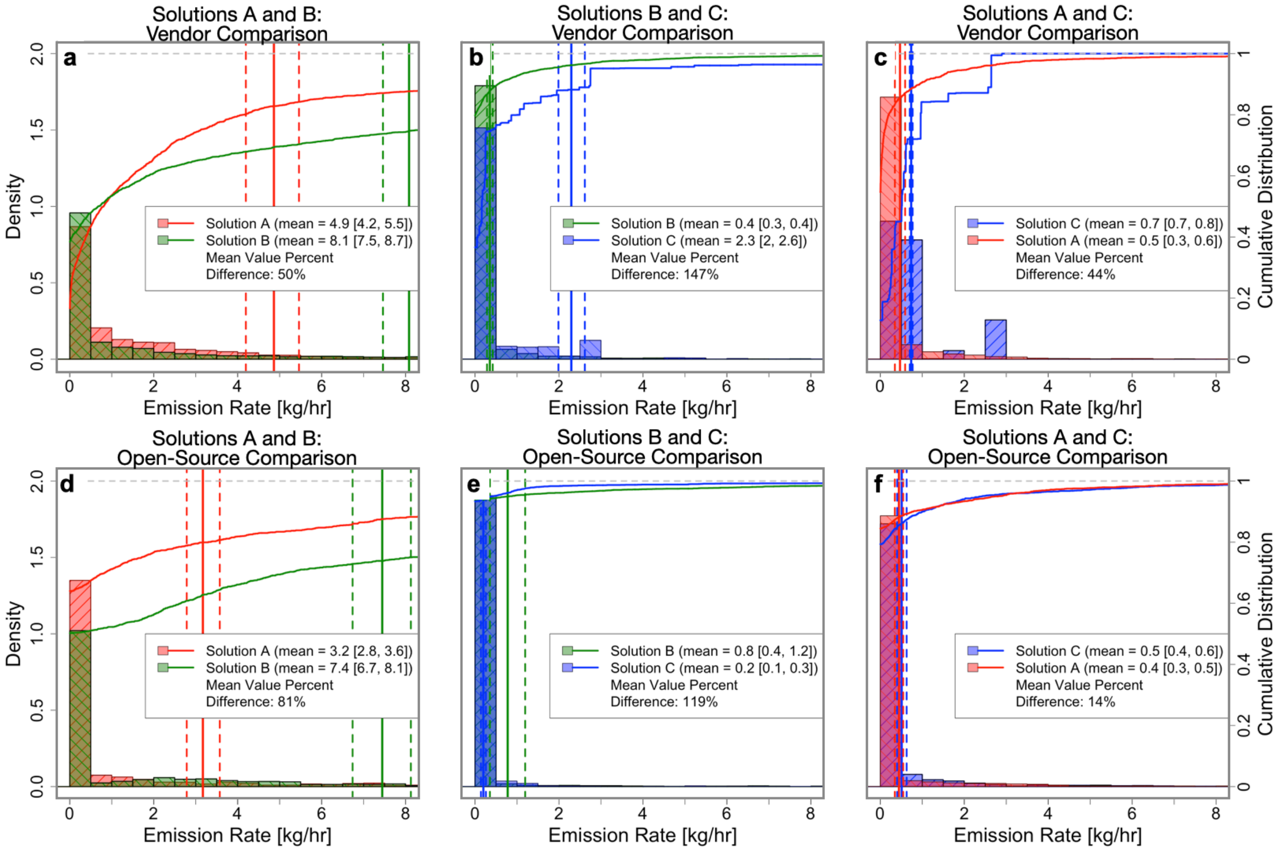


Figure S8: Distribution of emission rate estimates for each CMS solution pair, but only including the quantification estimates where the corresponding localization estimate was the same between CMS solutions. (a)-(c) show rate estimates provided by the CMS vendors, and (d)-(f) show rate estimates from the open-source DLQ algorithm applied to the raw concentration data from each CMS solution. Each subfigure uses data from the two oil and gas sites that have the two solutions installed. Solid lines show empirical cumulative distribution functions, solid vertical lines show distribution averages, and dashed vertical lines show 95% confidence intervals for the averages. Horizontal axes are restricted to [0, 8] kg/hr to show detail. Density is a scaled version of the counts in each bin such that each histogram has a unitary area.

Many of the distribution means are shifted up as compared to Figure 5 in the main manuscript, indicating that DLQ localization estimates are less likely to agree for smaller emissions. Additionally, the alignment between some solutions improved when compared to Figure 5 in the main manuscript, such as the B-C and A-C comparisons, while the alignment between others got worse, such as the A-B comparison. Whether or not the alignment improved after controlling for different localization estimates may be a function of the different site geometries or the number of remaining emission rate estimates.

Figure S9 shows QQ plots for the emission rate distributions shown in Figure S8 that only include the rate estimates where the corresponding localization estimates agreed between solutions. The RMSE for each comparison is shown in the top right of each subfigure. The RMSE is sensitive to outliers, so we also list the RMSE calculated after excluding the top 5% of the emission rate estimates to capture the bulk of the distribution. The axes are limited to [0, 20] kg/hr for clarity.

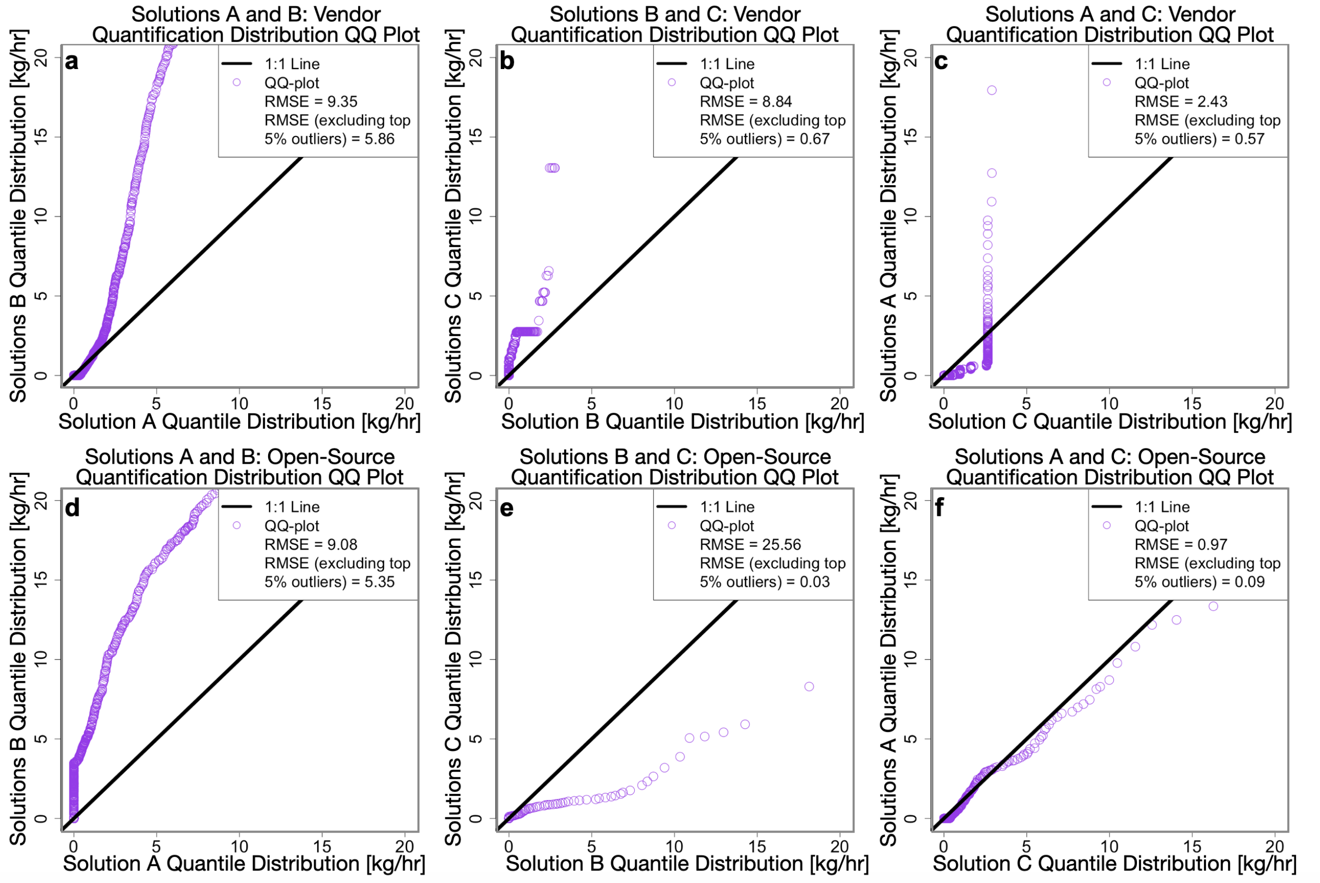


Figure S9: Quantile-quantile plots of the emission rate estimates from Figure S8 that are filtered to intervals where both solutions localize to the same emission source. The overall RMSE and the RMSE calculated after excluding the top 5% of the emission rate estimates is listed in the legend.

As with the histograms in Figure S8, the QQ plots show closer alignment in the B-C and A-C comparison, but worse alignment in the A-B comparison as compared to Figure 5 in the main text.

# References


[1] W. S. Daniels, M. Jia, and D. M. Hammerling, “Detection, localization, and quantification of single-source methane emissions on oil and gas production sites using point-in-space continuous monitoring systems,” *Elementa: Science of the Anthropocene*, vol. 12, p. 00110, Mar. 2024. https://doi.org/10.1525/elementa.2023.00110.
